# TRIM65/NF2/YAP1 Signaling Coordinately Orchestrates Metabolic and Immune Advantages in Hepatocellular Carcinoma

**DOI:** 10.1002/advs.202402578

**Published:** 2024-07-15

**Authors:** Zhixuan Bian, Chang Xu, Xiaoying Wang, Baohua Zhang, Yixuan Xiao, Li Liu, Shasha Zhao, Nan Huang, Fengjiao Yang, Yue Zhang, Shaobo Xue, Xiongjun Wang, Qiuhui Pan, Fenyong Sun

**Affiliations:** ^1^ Department of Laboratory Medicine Shanghai Children's Medical Center School of Medicine Shanghai Jiao Tong University Shanghai 200127 China; ^2^ Faculty of Medical Laboratory Science College of Health Science and Technology School of Medicine Shanghai jiao Tong University Shanghai 200025 China; ^3^ Shanghai Key Laboratory of Clinical Molecular Diagnostics for Paediatrics Shanghai 200127 China; ^4^ Department of Laboratory Medicine Shanghai Tenth People's Hospital of Tongji University Shanghai 200072 China; ^5^ Department of liver surgery Zhongshan hospital Fudan University Shanghai 200030 China; ^6^ Department of Central Laboratory Shanghai Tenth People's Hospital of Tongji University Shanghai 200072 China

**Keywords:** hepatocellular carcinoma, immune evasion, palmitic acid, TRIM65, uracil metabolism

## Abstract

Hepatocellular carcinoma (HCC) is one of the leading causes of cancer deaths worldwide. Significantly activated uridine nucleotide and fatty acid metabolism in HCC cells promote malignant proliferation and immune evasion. Herein, it is demonstrated that the tripartite motif 65 (TRIM65) E3 ubiquitin‐protein ligase, O‐GlcNAcylated via O‐GlcNAcylation transferase, is highly expressed in HCC and facilitated metabolic remodeling to promote the accumulation of products related to uracil metabolism and palmitic acid, driving the progression of HCC. Mechanistically, it is showed that TRIM65 mediates ubiquitylation at the K44 residue of neurofibromatosis type 2 (NF2), the key protein upstream of classical Hippo signaling. Accelerated NF2 degradation inhibits yes‐associated protein 1 phosphorylation, inducing aberrant activation of related metabolic enzyme transcription, and orchestrating metabolic and immune advantages. In conclusion, these results reveal a critical role for the TRIM family molecule TRIM65 in supporting HCC cell survival and highlight the therapeutic potential of targeting its E3 ligase activity to alter the regulation of proteasomal degradation.

## Graphical abstract

TRIM65, an E3 ligase, is O‐GlcNAcylated and highly expressed in hepatocellular carcinoma (HCC). TRIM65 promotes the accumulation of products related to uracil metabolism and palmitic acid via NF2/YAP1 signaling, orchestrating metabolic and immune advantages to drive the progression of HCC.

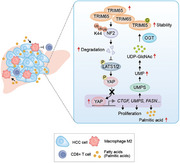

## 1 Introduction

Hepatocellular carcinoma (HCC), as one of the most common malignancies, is the third leading cause of cancer‐related deaths worldwide.^[^ ^1^ ^]^ Despite the fact that HCC treatment has made considerable advances with the comprehensive application of radiotherapy, chemotherapy, and immunotherapy, the 5‐year survival rate of patients with HCC is only ≈18%.^[^ ^2^ ^]^ Drug resistance, high risk of recurrence, and metastasis contribute to the poor prognosis.^[^ ^3^ ^]^ Therefore, it is urgent to elucidate the molecular mechanisms underlying the pathogenesis of HCC, and to develop novel reliable diagnostic and predictive biomarkers, so as to provide more effective solutions for HCC therapy.

Previous studies have revealed that metabolic reprogramming could satisfy the demands of HCC cells for survival and ensure advantages in growth.^[^ ^4^ ^]^ Nucleotide synthesis affects several important biological processes in various cancer cell types, and is therefore also responsible for uncontrolled proliferation, metastasis, immune evasion, and therapy resistance.^[^ ^5^, ^6^, ^7^ ^]^ However, there is a deficiency in regulation of the uridine monophosphate synthetase (UMPS), one of the most vital enzymes in the uracil de novo pathway, which transforms orotate and phosphoribosyl pyrophosphate into uridine monophosphate (UMP).^[^ ^8^ ^]^ Additionally, UDP‐N‐acetyl‐D‐galactosamine (UDP‐GlcNAc), a downstream product of the UMP metabolic pathway, is the only substrate for protein O‐GlcNAcylation (O‐GlcNAc).^[^ ^9^ ^]^ Our previous study reported that O‐GlcNAc significantly facilitates HCC malignancy.^[^ ^10^ ^]^ Thus, UMP metabolism plays a crucial role in the development of HCC and whether UMP depletion due to UMPS deficiency is an effective target for the treatment of HCC has not been examined.

To satisfy the nutrient demand for HCC cells, fatty acid oxidation is another major pathway besides glycolysis, which provides energy and metabolites for cell growth.^[^ ^11^ ^]^ As the liver is a most pivotal organ in lipid homeostasis, dysregulated fatty acid metabolism has been considered to drive HCC pathogenesis.^[^ ^12^, ^13^ ^]^ Palmitic acid (PA), a saturated fatty acid, is involved in the progression of HCC.^[^ ^14^ ^]^ Furthermore, PA‐modified albumin could selectively target polarized tumor‐associated macrophages (TAMs).^[^ ^15^, ^16^ ^]^ These data indicate that PA may function in the remodeling of the HCC immune microenvironment, and especially in macrophage polarization through metabolic adaptation, suggesting a prospective therapy targeting HCC.

The Neurofibromatosis Type 2 (NF2) gene that encodes the Mosein‐ezrin‐radaxin protein (Merlin) is an indispensable tumor suppressor; its depletion is prevalent in carcinogenesis and promotes the evolutionally conserved Hippo pathway to regulate homeostasis.^[^ ^17^ ^]^ NF2 activation phosphorylates transcriptional co‐activator Yes‐associated protein 1 (YAP1) that leads to cytoplasmic translocation and proteasomal degradation.^[^ ^18^ ^]^ Consequently, unphosphorylated YAP1 may enter the nucleus to activate the expression of oncogenes with transcription factors such as cyclic adenosine monophosphate response element binding protein (CREB).^[^ ^19^ ^]^ Several studies have identified NF2‐YAP1 signaling as one of the main factors in HCC.^[^ ^20^, ^21^ ^]^ However, the molecular mechanisms of NF2 expression in HCC remain poorly defined.

Tripartite motif (TRIM) proteins, one of the subfamilies of RING‐type E3 ubiquitin ligases, function as essential regulators of carcinogenesis.^[^ ^22^ ^]^ TRIM proteins are characterized by a coiled‐coil domain and a B‐box motif for zinc binding, as well as a RING finger domain, most of which serve as E3 ubiquitin ligases that participate in cell activity. Among these, TRIM65 has been identified to be involved in various pathogenesis of multiple tumor types.^[^ ^23^, ^24^, ^25^, ^26^ ^]^ However, the association between TRIM65 and HCC malignant progression in vivo has not yet been clarified. Therefore, the elucidation of the underlying mechanisms of TRIM65 promotion of HCC may provide a promising therapeutic strategy.

Here, we focus on the oncogenic role of TRIM65 in orchestrating metabolic and immune advantages in HCC using transgenic mouse models and identify TRIM65 as an essential promoter involved in the initiation and progression of HCC using two in situ models of HCC.

## 2 Results

### 2.1 TRIM65 was Up‐Regulated by OGT with a Poor Prognosis in HCC

**Figure 1:**
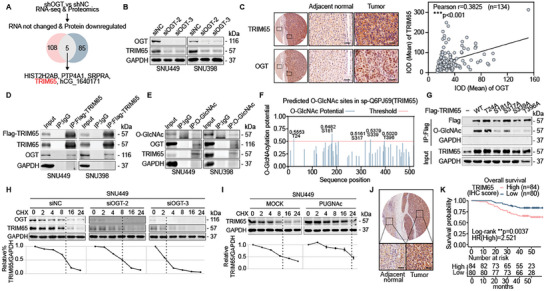
TRIM65 is upregulated by OGT in HCC. A) Venn diagram showing candidates whose protein levels downregulated with unchanging RNA levels in two HCC cells (red and blue) transfected with shNC or shOGT plasmids. B) Western blot (WB) analysis of OGT and TRIM65 in SNU449 and SNU398 cells transfected with siNC or siRNAs against OGT. C)Representative images of HCC tissue microarray with anti‐TRIM65 or anti‐OGT antibody staining and correlation analysis between OGT and TRIM65 protein levels in HCC tissues. Scale bar, 50 µm. D,E) Co‐IP assay performed using anti‐Flag and anti‐O‐GlcNAc antibodies in HCC cells. F) Predicted O‐GlcNAc sites in TRIM65. G) Co‐IP assay using anti‐Flag antibody in HEK‐293T cells transfected with Flag‐TRIM65 and O‐GlcNAc site mutants. H,I) Protein synthesis was blocked by cycloheximide (CHX) for the indicated times. The half‐life of OGT and TRIM65 in SNU449 cells transfected with siNC or siOGT or treated with PUGNAc were measured by WB. TRIM65 levels were normalized by GAPDH and the 0 h points were set to 100%. J) Representative image of TRIM65 IHC staining of HCC tissue microarray. Scale bar, 50 µm. K) Overall survival of HCC patients with high or low expression of TRIM65 using Kaplan–Meier method.

To screen the putative O‐GlcNAcylated proteins in HCC, RNA‐seq (Table S1, Supporting Information) and proteomics (Table S2, Supporting Information) were conducted using two HCC cell lines with the unique enzyme O‐GlcNAc transferase (OGT) deficiency. Given the O‐GlcNAcylation frequently increases the protein stability, we focused on the candidates whose protein levels were significantly downregulated, but RNA levels did not differ after OGT silencing. TRIM65 was identified among five candidates for further investigation (**Figure** **1**A). Consistent with the omics analysis, TRIM65 protein was obviously downregulated after OGT silencing (Figure 1B) but not the RNA level (Figure S1A, Supporting Information). We found a positive correlation between TRIM65 and OGT in paired HCC and adjacent normal tissues or in HCC tissue microarray staining (Figure 1C; Figure S1B,C, Supporting Information). Co‐immunoprecipitation (Co‐IP) assays were performed to demonstrate the interaction between TRIM65 and OGT (Figure 1D,E), supposing that TRIM65 was O‐GlcNAcylated by OGT. We then predicated the O‐GlcNAcylated sites in TRIM65,^[^ ^27^ ^]^ and five potential candidates including T24, S181, S317, S339, and T396 were identified (Figure 1F). Interestingly, Co‐IP demonstrated that more than one site (S181, S317, S339, and T396) in TRIM65 were O‐GlcNAcylated (Figure 1G). Furthermore, the stability of the TRIM65 protein appeared to be depleted when interfering with OGT expression (Figure 1H; Figure S1D, Supporting Information). In contrast, the half‐life of the TRIM65 protein in HCC cells under treatment with PUGNAc, an inhibitor of O‐GlcNAcase, was markedly prolonged (Figure 1I; Figure S1E, Supporting Information).

A pan‐cancer analysis based on multiple cancer tissue microarray showed that TRIM65 was selectively overexpressed in liver cancer (Figure S1F, Supporting Information) and we validated the up‐regulation of TRIM65 using the HCC tissue microarray and frozen tissues specimens (Figure 1J; Figure S1B,G,H, Supporting Information). More importantly, high expression of TRIM65 was significantly associated with a poor prognosis in patients with HCC (Figure 1K). Furthermore, the cox proportional hazards regression analysis verified the high expression of TRIM65 as an independent risk factor for HCC patients (HR = 2.370, p = 0.011, Figure S1I, Supporting Information). The receiver operating characteristic (ROC) curve and the area under curve (AUC) values showed that TRIM65 levels in tissues had diagnostic efficacy for HCC (AUC = 0.7487, *p* < 0.001, Figure S1J, Supporting Information). These findings revealed that TRIM65 was overexpressed in HCC and was correlated with a poor prognosis.

### 2.2 TRIM65 Promoted HCC Tumorigenesis in Mice

To illustrate the biological function of TRIM65, we transfected small interfering RNAs (siRNAs) targeting TRIM65 (siTRIM65) into HCC cells (SNU449 and SNU398) (Figure S2A, Supporting Information). The results showed that TRIM65 deficiency significantly inhibited proliferation with attenuated cell growth, colony formation capacity, and cell cycle arrest in G0‐G1 (Figure S2B,C,E, Supporting Information). Meanwhile, cell apoptosis was triggered by silencing TRIM65 expression (Figure S2D, Supporting Information). The xenograft model indicated that TRIM65 promoted HCC tumorigenesis in vivo (Figure S2F–I, Supporting Information). All of these data showed that TRIM65 played an important oncogenic role in HCC cells, which was consistent with a previous report.^[^ ^28^ ^]^

However, considering the complexity of the tumor microenvironment, it remained to be determined whether TRIM65 contributes to the pathogenesis of HCC. Therefore, mice with Trim65 expression conditionally knocked out (cKO) in hepatocytes were generated using the Cre/loxp system (Figure S3A, Supporting Information). We first obtained genetically homozygous offspring (Trim65^fl/fl^; Albumin‐Cre (Alb‐Cre) and Trim65^fl/fl^) after hybridization (Figure S3B,C, Supporting Information). There were no morphological or histological differences in the liver of mice with or without Trim65 cKO, and IHC staining revealed a similar normal proliferation ability (Figure S3H, Supporting Information). Compared to Trim65^fl/fl^, Trim65^fl/fl^; Alb‐Cre had barely any effect on liver weight or on the liver‐to‐body weight ratio (Figure S3D,E, Supporting Information). Furthermore, plasma levels of alanine aminotransferase (ALT) and aspartate transaminase (AST) were similar (Figure S3F,G, Supporting Information). All these data indicated that Trim65 cKO in hepatocytes would not have an impact on normal liver structure and function.

**Figure 2:**
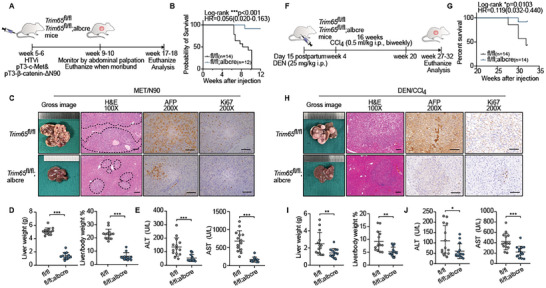
TRIM65 plays an oncogenic role in HCC mice models. A) Study design of the MET/N90 HCC model. B) Survival analysis of MET/N90 mice using Kaplan–Meier method. C) Gross image of livers, H&E, AFP and Ki67 staining in Trim65^fl/fl^; Alb‐Cre (*n* = 12) and Trim65^fl/fl^ (*n* = 14) mice treated with MET/N90. Scale bar, 100 µm. D,E) Liver weight, liver‐to‐body weight ratio, and ALT and AST levels of mice after MET/N90 induction. F) Study design of the DEN/CCl_4_ HCC model. G) Survival analysis of DEN/CCl_4_ mice using Kaplan–Meier method. H) Gross image of livers, H&E, AFP and Ki67 staining in Trim65^fl/fl^; Alb‐Cre (*n* = 14) and Trim65^fl/fl^ (*n* = 14) mice treated with DEN/CCl_4_. I,J) Liver weight, liver‐to‐body weight ratio, and ALT and AST levels of mice after DEN/CCl_4_ induction.

Subsequently, we constructed an in situ HCC model^[^ ^29^ ^]^ using Sleeping Beauty (SB) transposase along with pT3‐cMet and pT3‐ΔN90‐β‐catenin or equivalent saline by hydrodynamic tail vein injection (HTVi) in Trim65^fl/fl^ and Trim65^fl/fl^;Alb‐Cre mice respectively (MET/N90 for short) (**Figure** **2**A; Figure S3I, Supporting Information) and survival analysis revealed that Trim65 expression led to a poor prognosis implying a more severe tumor burden (Figure 2B). In particular, livers from Trim65^fl/fl^ mice representatively showed substantive multifocal disease with multiple tumor nodules, even intratumoral hemorrhage and bile‐filled vesicles in advanced stages, whereas livers from Trim65 cKO mice represented mild focal lesions (Figure 2C). Furthermore, alpha‐fetoprotein (AFP), ALT, and AST decreased significantly in the Trim65 cKO group combined with an apparently lower liver weight and liver‐to‐body weight ratio (Figure 2C–E), indicating a more serious liver injury in the presence of Trim65. To validate the oncogenic role of Trim65 in vivo, we established another HCC model by intraperitoneally injecting diethylnitrosamine (DEN) and carbon tetrachloride (CCl_4_) (DEN/CCl_4_) at indicated timepoints (Figure 2F). Consistently, Trim65 cKO mice showed remarkably prolonged survival and a suppression of HCC with less tumor nudes and abrogated malignant proliferation induced by DEN/CCl_4_ (Figure 2G,H). Furthermore, liver function in Trim65 cKO mice improved markedly, presenting lower levels of AFP, ALT, and AST (Figure 2H–J). Taken together, Trim65 promotes HCC tumorigenesis induced by MET/N90 or DEN/CCl_4_ and may serve as a promising target for HCC therapy.

### 2.3 TRIM65‐Induced Pathogenesis in HCC Relies on its E3 Ligase Activity

**Figure 3:**
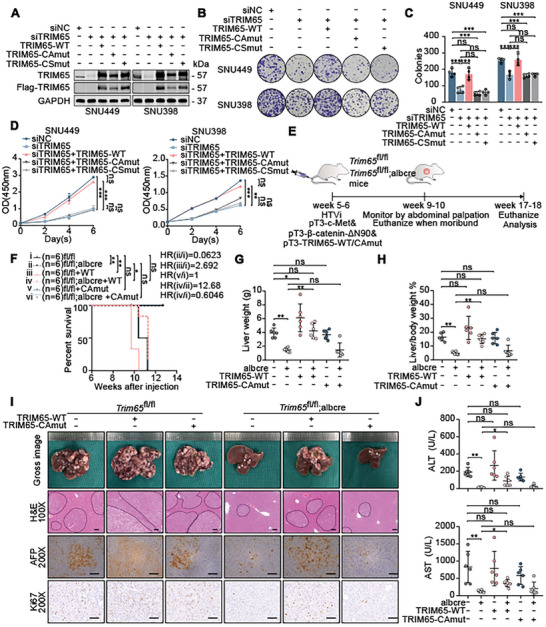
TRIM65 functions as an E3 ligase to promote the pathogenesis of HCC. A) The transfection efficiency of interfering or overexpression of TRIM65 mutants was measured by WB in HCC cells. B,C) Colony formation assays. D) CCK8 assays. E) Study design of TRIM65 rescue model. F) Survival analysis of MET/N90, MET/N90/TRIM65‐WT, and MET/N90/TRIM65‐CAmut in Trim65^fl/fl^;Alb‐Cre and Trim65^fl/fl^ mice (*n* = 6 for each group) using the Kaplan‐Meier method. G,H) Liver weight and liver‐to‐body weight ratio among the above groups. I) Gross image of livers, H&E, AFP, and Ki67 staining in the above groups. Scale bar, 100 µm. J) Plasma ALT and AST levels in mice.

We then evaluated whether the oncogenic role of TRIM65 was mediated by its E3 ligase activity. As TRIM65 has an RBCC (RING, B‐box and coiled‐coil) ubiquitin ligase domain,^[^ ^22^ ^]^ the first and second Cystine (Cys12 and Cys15) in the TRIM65 RING domain were mutated to Alanine or Serine (TRIM65‐CAmut and TRIM65‐CSmut, respectively) to destroy the E3 ligase activity^[^ ^30^ ^]^ and the two mutants were co‐transfected with siTRIM65 to HCC cells (**Figure** **3**A). In particular, the decreased proliferation ability induced by TRIM65 silencing was almost reversed by overexpression of wild‐type TRIM65 (TRIM65‐WT) but not of two mutants (Figure 3B–D), suggesting that TRIM65 contributes to proliferation based on its E3 ligase activity in HCC cells.

Furthermore, we co‐injected TRIM65‐WT or TRIM65‐CAmut plasmids into the MET/N90 model to rescue HCC carcinogenesis in vivo (Figure 3E). The efficiency of overexpression was detected by IHC using mouse liver tissues (Figure S3J, Supporting Information). Interestingly, TRIM65‐WT injection further exacerbated the progression of HCC and reversed the inhibition caused by Trim65 cKO, whereas the complement TRIM65‐CAmut showed no difference from the control treatment in terms of survival (Figure 3F). Furthermore, the burden of liver tumors was selectively increased in the group supplemented with TRIM65‐WT but not with TRIM65‐CAmut (Figure 3G,H). The higher expression of TRIM65‐WT aggravated the malignant degree of HCC of those with elevated levels of AFP, ALT, and AST that indicate abnormal liver function (Figure 3I,J). The abrogated HCC progression triggered by Trim65 cKO could not be rescued by TRIM65‐CAmut (Figure 3I,J). Collectively, these results revealed that the pathogenesis triggered by TRIM65 in HCC depends on its activity of E3 ligase.

### 2.4 TRIM65 Facilitated the Nuclear Translocation of YAP1

**Figure 4:**
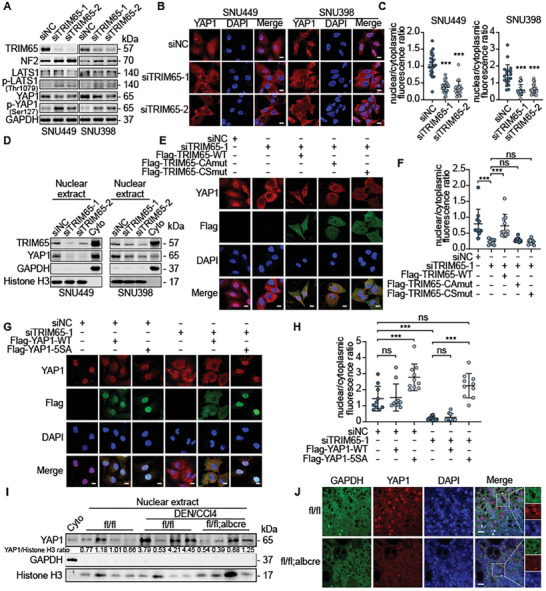
TRIM65 facilitates YAP1 accumulation in the nucleus. A) WB analysis of the NF2‐YAP1 signaling pathway in HCC cells. B) The cellular location of YAP1 assessed by IF. Scale bar, 10 µm. C) Nuclear cytoplasmic fluorescence ratios of YAP1. D) Nuclear and cytoplasmic extracts were analyzed by WB. GAPDH and Histone H3 were used as cytoplasmic and nuclear markers. E) Exogeneous Flag‐TRIM65 and YAP1 localization were detected by IF in TRIM65 knockdown or overexpressed SNU449 cells. Scale bar, 10 µm. G) The localization of exogenous Flag‐YAP1 and total YAP1 was detected by IF in SNU449 cells co‐transfected with siRNAs and YAP1‐WT or YAP1‐5SA plasmids. Scale bar, 10 µm. (F, H) YAP1 nuclear and cytoplasmic fluorescence ratio upon corresponding analysis of IF. I) WB showing the YAP1 expression of DEN/CCl_4_ mice liver tissues in nuclear and cytoplasmic extracts. J) IF assay of mice liver tissues from DEN/CCl_4_‐treated mice showing the localization of YAP1. GAPDH and DAPI were used as cytoplasmic and nuclear markers. Scale bar, 100 µm.

To elucidate the underlying mechanism of TRIM65 as a critical oncogene in HCC, we investigated the correlated signaling pathway.^[^ ^28^, ^31^ ^]^ TRIM65 had no effect on the expression of p53 and AXIN1 in HCC cells (Figure S4A, Supporting Information). Furthermore, we did not observe any alteration of β‐catenin nuclear localization with TRIM65 knockdown (Figure S4B, Supporting Information). Taking into account our previous work,^[^ ^10^, ^19^ ^]^ we evaluated whether TRIM65 had an impact on the YAP1 signaling pathway. Interestingly, NF2, a key tumor suppressor, responsible for activation of LATS1 (Large tumor suppressor homolog 1) and increased YAP1 phosphorylation, was significantly up‐regulated after silencing of TRIM65 expression (**Figure** **4**A). Consequently, phospho‐LATS1 (p‐LATS1) and phospho‐YAP1 (p‐YAP1) were elevated, while total LATS1 and YAP1 decreased slightly (Figure 4A). Similarly, YAP1 was significantly downregulated in Trim65 cKO groups with DEN/CCl_4_ or MET/N90 treatment (Figure S4G, Supporting Information), implying that TRIM65 is involved in the regulation of the YAP1 signaling pathway in HCC cells. Furthermore, we used an immunofluorescence (IF) assay to identify the subcellular location of YAP1 in HCC cells. The nuclear translocation of YAP1 was markedly reduced when TRIM65 was deficient (Figure 4B,C). Furthermore, YAP1 levels in the nuclear extracts were obviously reduced by silencing TRIM65 expression (Figure 4D), indicating that TRIM65 promoted the nuclear translocation of YAP1. In particular, we confirmed that nuclear extracts and the nuclear localization signal of YAP1 from the liver tissues of Trim65 cKO mice were decreased in DEN/CCl_4_ and MET/N90 HCC models (Figure 4I,J; Figure S4H,I, Supporting Information), which was consistent with the delayed HCC progression.

The results showed that nuclear localization of YAP1 could be rescued by retrieving TRIM65‐WT but not by its mutants in HCC cells (Figure 4E,F; Figure S4C,E, Supporting Information), demonstrating that TRIM65‐induced ubiquitylation resulted in activation of YAP1. Furthermore, overexpression of YAP1‐5SA, an activated mutant of YAP1 with deficient phosphorylation, could reverse the cytoplasmic localization caused by silencing of TRIM65 (Figure 4G,H; Figure S4D,F, Supporting Information), indicating that TRIM65 participates in the upstream activation of YAP1 in HCC cells. Taken together, we determined that TRIM65 facilitates nuclear translocation of YAP1 and activates the YAP1 signaling pathway in HCC.

### 2.5 TRIM65 Mediates NF2 Ubiquitylation at the K44 Residue

**Figure 5:**
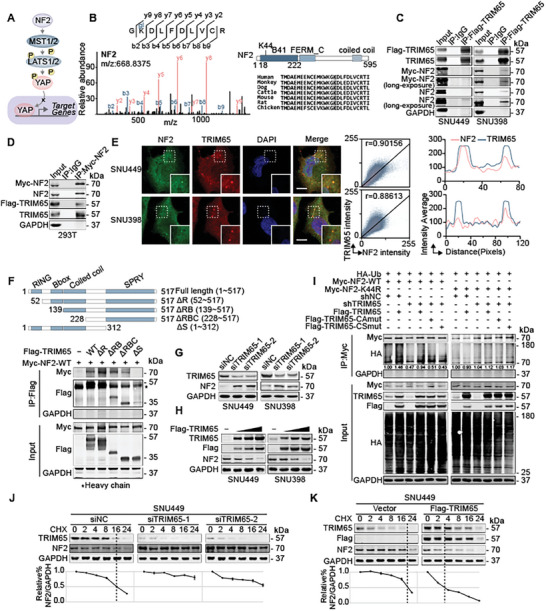
TRIM65 mediates the ubiquitylation of NF2 at the K44 residue. A) Schematic illustration of the NF2/YAP1 signaling pathway. B) The NF2 K44 site was identified by ubiquitome analysis and was evolutionarily conserved. C) IP assays using anti‐FLAG antibody were analyzed by WB in HCC cells co‐transfected with the Myc‐NF2 and Flag‐TRIM65 plasmids. D) Myc‐NF2 was immunoprecipitated by anti‐MYC antibody in HEK‐293T cells. WB detected endogenous and exogenous TRIM65 binding with NF2. E) IF assays were performed in HCC cells using anti‐NF2 and anti‐TRIM65 antibodies. The intensity correlation was analyzed by Image J software. Scale bar, 5 µm. F) Top, schematic diagram of TRIM65 truncated mutants. Bottom, interactions between the NF2 and TRIM65 truncations were detected by IP‐WB assays. G) WB analysis of TRIM65 and NF2 in HCC cells transfected with siNC or siTRIM65. H) WB analysis of TRIM65 and NF2 in HCC cells transfected with control or increased concentration of Flag‐TRIM65 plasmids. I) IP assays using anti‐MYC antibody were performed in HEK‐293T cells with indicated transfection vectors and analyzed by WB. J,K) Protein synthesis was blocked by CHX for the indicated times. The half‐life of NF2 and TRIM65 in SNU449 cells with the indicated treatments were measured by WB. NF2 levels were normalized by GAPDH and the 0 h points were set to 100%.

To identify the critical substrates ubiquitylated by TRIM65 in the YAP1 signaling pathway, we performed mass spectrometry to determine potential downstream targets. NF2, an inhibitor of the Hippo‐YAP1 signaling pathway, was ubiquitylated by TRIM65 at lysine‐44 (K44) residue (**Figure** **5**A,B). Subsequently, co‐IP and IF assays were performed to investigate the interaction between NF2 and TRIM65 (Figure 5C–E). To clarify the specific binding region in TRIM65, we constructed different truncated mutants of TRIM65 with deficiency of the RING (ΔR), RING & B box (ΔRB), RING & B box & coiled‐coil domain (ΔRBC) and SPRY (ΔS) domain deficiency, respectively (Figure 5F). Of these, the interaction between the NF2 and TRIM65ΔS mutant was selectively decreased (Figure 5F), suggesting that TRIM65 binds to NF2 mediated by its SPRY domain. As expected, interfering TRIM65 expression markedly increased the levels of NF2 protein, whereas overexpression of TRIM65 was negatively related to the NF2 levels in a dose‐dependent manner (Figure 5G,H). To clarify that the NF2 K44 residue was ubiquitylated by TRIM65, we further mutated the single lysine to arginine (K44R) in NF2 to disrupt ubiquitylation. Consequently, weaker ubiquitylation of NF2 was observed after overexpression of the K44R mutant (Figure 5I). TRIM65‐CAmut or TRIM65‐CSmut could not recover ubiquitylation of NF2 (Figure 5I). After cell proteasome inhibitor, MG132, treatment, the protein level of NF2 was upregulated in TRIM65 overexpressed cells (Figure S4J, Supporting Information). Furthermore, TRIM65 negatively regulated the stability of the NF2 protein (Figure 5J–K; Figure S4K,L, Supporting Information). Collectively, these results revealed that TRIM65 mediated the ubiquitylation of NF2 at the K44 residue, accelerating protein degradation.

### 2.6 TRIM65 Stimulated Uracil Metabolism by Activating UMPS Transcription through YAP/CREB

**Figure 6:**
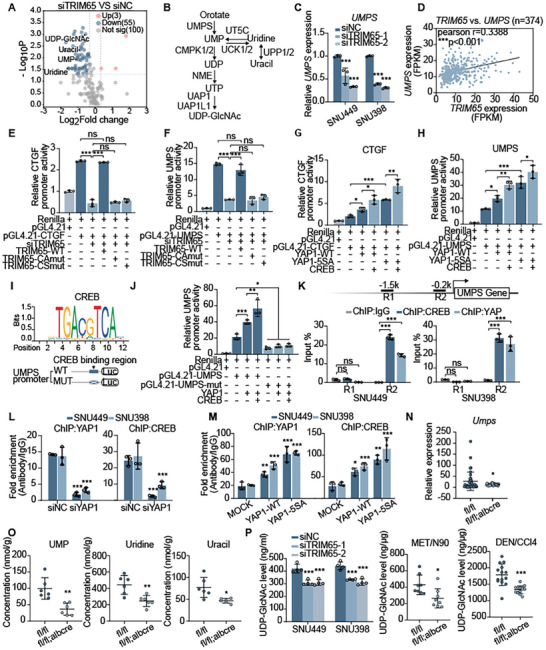
TRIM65 promotes uracil metabolism by upregulating UMPS transcription via YAP1 activation. A) Volcano plot of differentially‐regulated metabolites between siTRIM65 and siNC groups in metabolomic analysis. B) Summary of metabolites in uracil metabolism. C) Relative expression of UMPS mRNA in HCC cells with TRIM65 knockdown. D) Correlation analysis representing the expression of TRIM65 and UMPS in the TCGA LIHC database. E–H,J) The relative activity of the CTGF and UMPS promoter was measured by a dual‐luciferase reporter assay in HEK‐293T cells. I) Motif analysis of the UMPS promoter containing the CREB binding region. K–M) ChIP assays were performed with control IgG, CREB, or YAP1 antibodies as indicated. N) Relative expression of Umps mRNA in Trim65^fl/fl^; Alb‐Cre and Trim65^fl/fl^ mice liver tissues. O) The concentration of UMP, uridine, and uracil in mice detected by MS. P) The UDP‐GlcNAc level was examined in HCC cells and mice models (MET/N90 and DEN/CCl_4_).

Since we determined that TRIM65 was involved in the regulation of the NF2‐YAP signaling pathway, we then elucidated the downstream targets regulated by the activation of YAP1. Considering YAP1 functions as a sensor in the tumor microenvironment, we performed a metabolic analysis, which showed that uracil metabolism was significantly enriched with UMP, uracil, uridine, and UDP‐GlcNAc decreasing after TRIM65 knockdown (**Figure** **6**A). Therefore, we verified the expression of a panel of enzymes in uracil metabolism associated with TRIM65 levels (Figure 6B; Figure S5A,B, Supporting Information). In particular, UMPS, a differential enzyme in the primary step of uracil de novo synthesis that transforms the orotate into UMP, was selected for further investigation. As expected, UMPS expression was markedly decreased by TRIM65 deficiency in HCC cells (Figure 6C), which was consistent with downstream YAP1 targets such as cysteine‐rich angiogenic inducer 61 (CYR61),^[^ ^32^ ^]^ connective tissue growth factor (CTGF),^[^ ^33^ ^]^ and G1/S‐specific cyclin‐D1 (CCND1)^[^ ^34^ ^]^ (Figure S5C, Supporting Information). Analysis of TCGA database also indicated a significant positive correlation between the two proteins (Figure 6D). A dual‐luciferase assay was used to evaluate the transcriptional activity of UMPS, which was selectively activated by TRIM65‐WT but not by TRIM65‐CAmut or TRIM65‐CSmut, and showed similar changes in CTGF transcription. Overall, the results implied that TRIM65 accelerated UMPS transcription relying on its E3 ligase activity (Figure 6E,F). Additionally, the complement of YAP1‐5SA or CREB, a transcriptional coactivator of YAP1,^[^ ^35^ ^]^ further promoted UMPS transcription evaluated indirectly by changes in CTGF expression, indicating that YAP1 and CREB were involved in UMPS transcriptional activation (Figure 6G,H). Subsequently, the CREB binding region in the UMPS promoter was identified and was used to engineer its deletion mutant (UMPS‐mut) to better clarify its role in transcriptional regulation (Figure 6I,J). ChIP (Chromatin Immunoprecipitation)‐qPCR was performed and CREB or YAP1 binding to the UMPS promoter was validated (Figure 6K). The interaction appeared to be reduced after silencing of YAP1, while forced expression of YAP1 significantly improved the conjunction (Figure 6L,M). Relatively, YAP1‐5SA could further intensify the binding of CREB and YAP1 to the UMPS promoter (Figure 6M). From these data, we preliminarily concluded that TRIM65 promoted UMPS transcription by activating YAP1/CREB.

We then explored the expression of Umps in Trim65^fl/fl^ and Trim65^fl/fl^;Alb‐Cre mice liver tissues. Consistently, the Trim65 cKO group exhibited lower Umps expression compared to the control group (Figure 6N), which was similar to Ctgf, Ccnd1, and Cyr61 (Figure S5D, Supporting Information). We further performed a mass spectrometry analysis targeting uracil metabolism using mice liver tissues. The results showed that representative uracil metabolites, including UMP, uridine, and uracil, appeared to decrease in the liver of Trim65 cKO mice (Figure 6O). Given that uracil metabolism is essential for the synthesis of UDP‐GlcNAc, a pivotal substrate donor in O‐GlcNAcylation, we thus evaluated the levels of UDP‐GlcNAc in HCC cells and mice liver tissues. As expected, the level of UDP‐GlcNAc was significantly decreased both in TRIM65‐deficient HCC cells and in liver tissues of Trim65 cKO mice after MET/N90 or DEN/CCl_4_ treatment (Figure 6P).

Taken together, TRIM65 stimulates uracil metabolism by enhancing UMPS transcription via YAP1‐CREB coactivators in HCC, in turn composing a positive feedback loop between TRIM65 and O‐GlcNAcylation in HCC aggravation.

### 2.7 TRIM65 Contributed to an Immunosuppressive Tumor Microenvironment in HCC by Increasing Palmitic Acid Levels

**Figure 7:**
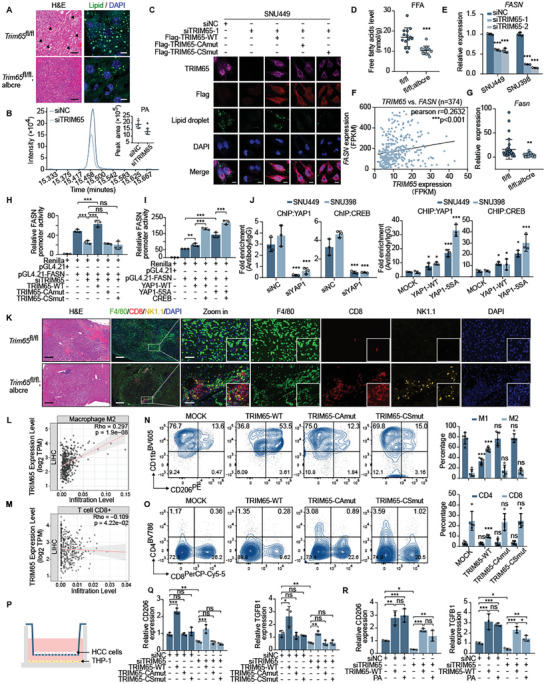
TRIM65 promotes the tumor immunosuppressive microenvironment in HCC by accumulating palmitic acids. A) H&E staining and IF assay targeting lipids in mice liver tissues. B) MS analysis of PA in SNU449 cells transfected with siNC or siTRIM65. C) The localization of TRIM65, Flag, and lipid droplets visualized by IF assay in SNU449 cells with indicated treatments. D) Free fatty acids in mice liver tissues. E,G) Relative expression of FASN detected by RT‐qPCR in HCC cells with indicated transfection and in mice liver tissues. F) Correlation analysis between FASN and TRIM65 expression using TCGA datasets. H,I) The relative activity of the FASN promoter was measured by a dual‐luciferase reporter assay in HEK‐293T cells with indicated transfection. J) ChIP assays were performed with IgG, CREB, and YAP1 antibodies followed by RT‐qPCR targeting the FASN promoter. K) H&E staining and IF assay targeting F4/80, CD8, and NK1.1 in mice liver tissues. L,M) Correlation analysis between TRIM65 expression and infiltration of M2 macrophages or CD8+ T cells. N,O) The macrophages and T cells from the liver tissues of mice were detected by flow cytometry after liver in‐situ injection with Hepa1‐6 cells, which were transfected with empty vector, TRIM65‐WT, CAmut, and CSmut. P) Diagram of the co‐cultured system. Q,R) Relative expression of CD206 and TGFB1 in co‐cultured THP‐1 cells detected by RT‐qPCR. SNU449 cells were pretreated with PA at a concentration of 400 µm for 24 h in R).

Severe hepatic steatosis in mice liver tissues were almost entirely reversed by Trim65 cKO in HCC in‐situ mice models (**Figure** **7**A), suggesting that Trim65 may participate in the regulation of lipid metabolism during pathogenesis. Metabolomics analysis revealed that PA and oleic acid, two main components of free fatty acids (FFA), were significantly decreased after TRIM65 knockdown (Figure 7B; Figure S6A, Supporting Information), indicating that TRIM65 influenced FFA accumulation in HCC cells. Consequently, lipid droplets, a form of intracellular lipid storage, were reduced when TRIM65 was deficient and could not be reversed in the presence of TRIM65‐CAmut or CSmut (Figure 7C; Figure S6B, Supporting Information). Similarly, FFA was obviously down‐regulated in the liver tissues of Trim65 cKO mice (Figure 7D). To clarify the underlying mechanism of the alteration, we evaluated the expression of FASN (Fatty acid synthase) in HCC cells and liver tissues of mice. As expected, FASN was significantly reduced by silencing of TRIM65 in vitro and in vivo (Figure 7E,G), and a positive correlation between FASN and TRIM65 was observed according to TCGA data (Figure 7F). Interestingly, FASN transcription was selectively activated by TRIM65‐WT (Figure 7H) and YAP1‐WT rescue, which increased further when YAP1‐5SA and CREB were supplemented (Figure 7I). Consistently, ChIP‐qPCR confirmed the interaction of YAP1 and CREB with the FASN promoter region in HCC cells (Figure 7J). Herein, we identified FASN as another downstream target of TRIM65 through YAP1/CREB‐mediated transcription activation, thus regulating lipid metabolism in HCC.

Given the association between intratumor steatosis and the tumor immunosuppressive microenvironment in HCC,^[^ ^36^ ^]^ we then examined the alteration of the tumor immune microenvironment induced by TRIM65. In particular, IF staining of murine liver tissues from the in‐situ HCC model revealed that Trim65 cKO induced CD8+ T cells and NK cells infiltration, which may result in considerable anti‐tumorigenic effects (Figure 7K). We performed a flow cytometric analysis using liver tissues of mice after MET/CAT treatment to verify the infiltration of immunocytes induced by Trim65 cKO. CD8+ T cells, M1 macrophages, and NK cells increased significantly when Trim65 was eliminated, while M2 macrophages appeared to be reduced by Trim65 cKO (Figure S6C–H, Supporting Information). Furthermore, the expression of TRIM65 was positively correlated with M2 macrophage infiltration and negatively correlated with CD8+ T cell infiltration according to the analysis of the TIMER database^[^ ^37^ ^]^ (Figure 7L,M), indicating that Trim65 contributed to the exhausted tumor immune microenvironment in HCC. We established an HCC model through in‐situ injection of mouse HCC cells (Hepa1‐6) transfected with TRIM65‐WT, CAmut, CSmut, or its control. Similarly, only forced expression of TRIM65‐WT could increase the infiltration of M2 macrophages and down‐regulate infiltration of CD8+ T cells, M1 macrophages, and natural killer (NK) cells (Figure 7N‐O; Figure S6I, Supporting Information), demonstrating that TRIM65 induced a tolerant immune microenvironment in HCC relying on its E3 ligase activity. Furthermore, human monocytic leukemia cells (THP‐1) and HCC cells were co‐cultured to induce macrophage polarization in vitro (Figure 7P). As expected, the expression of M2 macrophage markers, CD206, transforming growth factor beta‐1 (TGFB1), CD163, and interleukin‐10, was apparently reduced by silencing of TRIM65 expression, whereas mutants lacking E3 ligase did not reverse the suppression of M2 polarization induced by TRIM65 deficiency. Both TRIM65‐WT overexpression and PA treatment could recover M2 polarization (Figure 7Q,R; Figure S6J–N, Supporting Information), suggesting that TRIM65 is involved in M2 polarization partially through PA accumulation.

### 2.8 TRIM65 Served as a Pivotal Oncogene through the Activation of YAP in HCC

**Figure 8:**
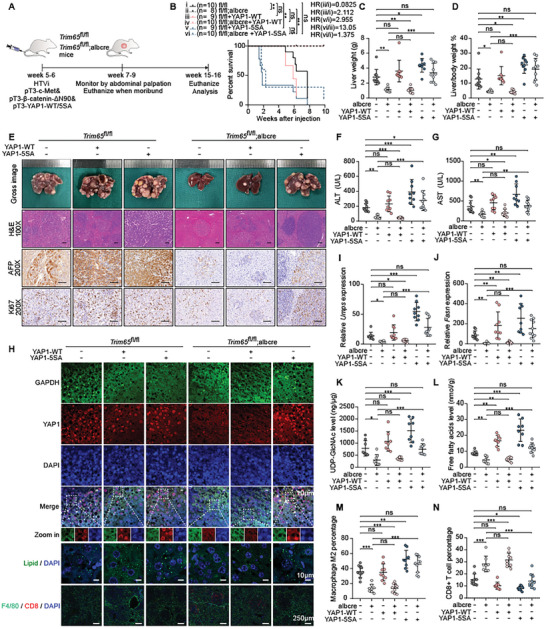
TRIM65 functions in carcinogenesis by over‐activating YAP1 in HCC mice model. A) Study design of YAP1 rescue model. B) Survival analysis of indicated treatments using the Kaplan–Meier method. C,D) Liver weight and liver‐to‐body weight ratio across the above groups. E) Gross image of livers, H&E, AFP, and Ki67 staining in the above groups. Scale bar, 100 µm. F,G) The plasma levels of ALT and AST in mice. H) IF staining of YAP1, GAPDH, lipid, F4/80, and CD8. Scale bar, as indicated in the figures. I,J) Relative expression of Umps and Fasn of Trim65 cKO in liver tissues of indicated groups. K,L) UDP‐GlcNAc and FFA levels in liver tissues of mice with the indicated treatments. M,N) Infiltration of M2 macrophages and CD8+T cells in mouse liver tissues with the indicated treatments.

To verify whether the malignant phenotype induced by TRIM65 was responsible for abnormal activation of YAP1, we transfected YAP1‐WT or YAP1‐5SA plasmids into the MET/N90 models of Trim65^fl/fl^ and Trim65^fl/fl^;Alb‐Cre mice respectively (**Figure** **8**A). The results showed that the complement of YAP1‐WT had little effect on survival, while injection of YAP1‐5SA could dramatically reverse the inhibition caused by Trim65 cKO and contributed to a similar shorter lifespan in both groups (Figure 8B). Similarly, YAP1‐5SA but not YAP1‐WT recovered the greatest tumor burden in Trim65 cKO mice, with a significantly elevated liver weight and liver‐body weight ratio (Figure 8C,D). Importantly, histological analysis revealed that most of the liver tissue was replaced by marked macroscopic tumors with multiple lesions in Trim65^fl/fl^ mice or mice under YAP1‐5SA treatment. Furthermore, Trim65 cKO delayed the progression of HCC even with YAP1‐WT induction, implying that Trim65 functions in carcinogenesis via YAP1 overactivation (Figure 8E). The staining of AFP and Ki67 in liver tissues combined with the level of ALT and AST in plasma was also consistent with the progressive stage of HCC (Figure 8E–G). Consequently, although injection of YAP1‐WT or YAP1‐5SA could obviously up‐regulate the total levels of YAP1, phosphorylated YAP1 and NF2 also increased, which was accompanied by considerably reduced levels of total YAP1 in liver tissues of Trim65 cKO (Figure S7A, Supporting Information). Furthermore, the tissue IF assay confirmed that YAP1 accumulation in the nucleus could be rescued in Trim65 cKO liver tissues after YAP1‐5SA expression was restored (Figure 8H), further demonstrating that Trim65 promoted nuclear translocation of YAP1 by upregulating its phosphorylation.

We then measured the expression of Umps, Fasn, and YAP1 downstream targets at the transcriptional level. Consequently, all were significantly downregulated by Trim65 deficiency and increased dramatically under YAP1‐5SA treatment in mice liver tissues (Figure 8I,J; Figure S7B, Supporting Information). As critical downstream metabolic pathways are mediated by TRIM65, the levels of UDP‐GlcNAc and FFA levels were assessed. Consistently, Trim65 cKO apparently decreased the levels of UDP‐GlcNAc and FFA along with the accumulation of lipid droplets, while YAP1‐5SA reversed the reduction and further reinforced these effects (Figure 8H,K,L). Meanwhile, we evaluated immunocyte infiltration in mice liver tissues. Similarly, Trim65 cKO reduced M2 macrophage infiltration, which was considerably increased by YAP1‐5SA rescued, and was opposite to CD8+ T cell infiltration (Figure 8H,M,N).

In summary, we demonstrated that TRIM65 serves as a central oncogene through the activation of YAP1 and up‐regulation of UDP‐GlcNAc and FFA levels, thus promoting the tumor immunosuppressive microenvironment in HCC.

## 3 Conclusion

The specific function of TRIM65 in HCC remains poorly defined. In this study, we first identified the potent pro‐carcinogenic role of TRIM65 in HCC through a transgenic mice model, which was remarkably associated with abnormal activation of uracil and FFA metabolism.

Metabolism reprogramming has been suggested as a new hallmark to rationalize the complexity of tumorigenesis.^[^ ^38^ ^]^ However, it remains to be thoroughly investigated whether and how key enzymes or intermediates play a key role in HCC tumorigenesis and progression. Purine and pyrimidine nucleotides are synthesized mainly by de novo and salvage pathways and require integrated metabolism of non‐essential amino acids, ribose, and one‐carbon donors. Nucleotide synthesis inhibitors have already been available as antineoplastic agents for cancer therapy and several enzymes relevant to pyrimidine metabolism are clinically approved as treatment options.^[^ ^8^ ^]^ Here, we found that TRIM65 could up‐regulate numerous metabolites in the uracil pathway, which served as nucleotides and as an energy source for cellular metabolism, promoting the rapid proliferation of HCC cells. Among these, UDP‐GlcNAc, a downstream product of UMP metabolism and the only substrate for intracellular O‐GlcNAcylation, showed a significant positive correlation with TRIM65. We found that TRIM65 could undergo O‐GlcNAcylation by OGT, further enhancing protein stability and forming a positive feedback pathway to accelerate HCC malignancy. These results showed that the dependence of HCC on nucleotide biosynthesis could be a crucial targetable metabolic vulnerability.

The incidence of nonalcoholic fatty liver disease and nonalcoholic steatohepatitis is increasing rapidly and fatty acid metabolic disorders have become a major risk factor for the development of HCC.^[^ ^39^ ^]^ In particular, a recent study showed that PA promoted lipid accumulation in HCC cells and induced the switching of immunosuppressive phenotypes of co‐cultured macrophages and fibroblasts,^[^ ^36^ ^]^ which linked cancerous steatosis and immunotherapy‐susceptible microenvironment in HCC. We found that TRIM65 significantly increased PA levels and that the overabundance of intracellular PA could be secreted into the tumor microenvironment, promoting the switching of TAM to M2 macrophages, and inhibiting the recruitment of CD8+ T cells, which suppressed the immunocidal effect, thus promoting malignant progression of HCC. These findings are consistent with a previous report indicating that steatotic HCC exhibits an immunoenriched but immunoexhausted tumor microenvironment.^[^ ^36^ ^]^ However, additional studies are needed to elucidate in detail how FFAs such as PA induce polarization of M2 macrophages.

**Figure 9:**
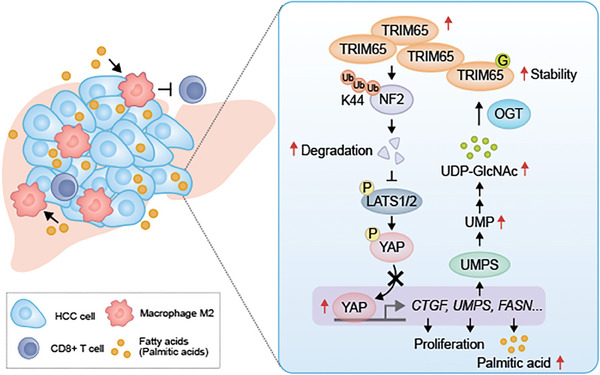
Diagram of the role of TRIM65 in HCC.

TRIM65, as a member of the TRIM family, can promote the ubiquitylation and degradation of target proteins, thus negatively regulating protein expression. Previous studies have reported that TRIM65 contributes to the activation of the β‐catenin signaling pathway by ubiquitylation of axis inhibition protein 1 (Axin1) in HCC,^[^ ^28^ ^]^ which is not consistent with the results of our study (Figure S4A, Supporting Information). We showed that TRIM65 was essential for liver tumorigenesis driven by MET/CAT or DEN/CCl_4_ in this study. Mechanistically, high‐expression of TRIM65 mediated the ubiquitylation of NF2 at the K44 residue toward degradation. As an important protein upstream of the Hippo signaling pathway, NF2 stimulates the accumulation of mammalian STE20‐like protein kinase 1/2 (MST1/2) and LATS1/2 kinases by forming a scaffold complex, after which MST1/2 activates LATS1/2 through phosphorylation, leading to inactivation of YAP1.^[^ ^17^ ^]^ In the present study, TRIM65 caused ubiquitylated degradation of NF2; thus, inducing nuclear translocation of YAP1 and remodeling of metabolic homeostasis. As a transcriptional coactivator, YAP1 activated UMPS and FASN transcription, resulting in exuberant intracellular UMP and PA synthesis, which promoted HCC cell proliferation and enrichment of immunosuppressive macrophage, facilitating HCC malignancy (**Figure** **9**). Drugs that inhibit TRIM65 activity by blocking E3 ligase activity or suppressing interaction with substrates in the C‐terminal structural domain may be clinically useful. A deeper understanding of the ubiquitin proteasome system and the activity of TRIM65 E3 ligase may provide effective targets for novel cancer‐targeted HCC therapies.

In HCC, TRIM65 was significantly overexpressed via O‐GlcNAcylation by OGT and mediated ubiquitylation of NF2 at the K44 residue toward degradation as an E3 ligase, inhibiting the phosphorylated activation of LATS1/2 resulting in the accumulation of YAP1 in the nucleus, thus promoting the transcription of downstream targets, including CTGF, UMPS and FASN. UMPS enhanced uracil metabolism, and in turn, upregulated O‐GlcNAcylation substrate UDP‐GlcNAc levels to compose a positive feedback loop in HCC aggravation. Conversely, FASN increased the level of FFA and in particular, that of PA, to increase the polarization of M2 macrophages and inhibit the infiltration of CD8+ T cells, thus promoting HCC progression through the construction of an exhausted tumor immune microenvironment.

## 4 Experimental Section

### Patients with HCC and Clinical Specimens

Paired HCC tissues and adjacent para‐tumor tissues were obtained from Shanghai Tenth People's Hospital with the approval of the ethics committees and the written consent of each participant. After surgery, tissue samples were immediately snap‐frozen in liquid nitrogen for further tests. HCC tissue microarrays with available clinical information were obtained from Zhongshan Hospital Affiliated to Fudan University. Multiple cancer tissue microarray (24 cases of multiple types of cancer with matched or matched normal controls) was obtained from US Biomax (Cat#MC481).

### Cell Lines and Cell Culture

The SNU‐398, SK‐Hep‐1, Huh6, Hepa1‐6, and HEK‐293T cell lines were purchased from the Bank of the Type Culture Collection of the Chinese Academy of Sciences (Shanghai, China). The SNU‐449 and THP‐1 cell lines were obtained from the BeNa Culture Collection (Beijing, China). SNU‐449 and SNU‐398 were cultured in RPMI‐1640 (Gibco, Cat# C11875500BT) and SK‐Hep‐1, Huh6, Hepa1‐6, and HEK‐293T cells were maintained in DMEM (Gibco, Cat#C11995500BT) supplemented with 10% fetal bovine serum (FBS, Gibco, Cat#10 099 141) and 1% penicillin‐streptomycin solution (PS, NCM Biotech, Cat#C100C5) in a humidified incubator containing 5% CO_2_ at 37 °C. The THP‐1 cell line was maintained in RPMI‐1640 medium supplemented with 10% FBS, 1% PS, and 0.05 mm β‐mercaptoethanol (Sigma‐Aldrich, Cat#M3148).

### Plasmids, siRNAs, and Cell Transfection

The plasmids were designed and prepared by Ke Lei Biological Technology (Shanghai, China). Small interfering RNAs (siRNAs) were purchased from GenePharma (Shanghai, China) and both were transfected using the Lipofectamine 2000 reagent (Invitrogen, Cat#11 668 030) according to the manufacturer's instructions. To construct stable knockdown or overexpression cell lines, lentiviruses were generated from HEK‐293T cells by co‐transfection of the indicated plasmids and two packing vectors (PsPAX2 and pMD2.G). HCC cells were infected with lentivirus harvested at 24 and 48 h by polybrene (Santa Cruz Biotech, Cat#sc‐134220) treatment for 12–24 h, after which the positively infected cells were collected with 2 µg mL^−1^ puromycin (Invitrogen, Cat#A1113803) treatment for two weeks. SiRNAs used in this study are listed in Table S3 (Supporting Information).

### Animal Experiments

All procedures related to animal studies were performed in accordance with the guidelines approved by the Animal Research Ethics Committee of Shanghai Tenth People's Hospital (SHDSYY‐2023‐2301). Trim65^fl/−^ and Alb‐Cre C57BL/6J mice were obtained from the Shanghai Model Organisms Center (Shanghai, China). Then Trim65^fl/fl^ mice were hybridized with Alb‐Cre mice to acquire Trim65 cKO mice (Trim65^fl/fl^; Alb‐Cre).

To induce hepatocellular carcinogenesis, 25 mg kg^−1^ DEN (Sigma‐Aldrich, Cat# 73 861) was injected intraperitoneally into day 15 postpartum mice followed by continuous injections of 0.5 mg kg^−1^ CCl_4_ biweekly for ≈16 weeks. Six or seven months after DEN injection, the mice were euthanized and liver tissues were collected. Oncogenic systems composed of pT3‐EF1α‐c‐MET, pT3‐EF1α‐ΔN90‐β‐catenin, and transposase plasmids pCMV‐T7‐SB100 in 2 mL saline solution were quickly injected into the tail vein within 7 s to establish the in situ HCC mice model. For rescue experiments, pT3‐EF1α‐TRIM65‐WT, pT3‐EF1α‐TRIM65‐CAmut, pT3‐EF1α‐YAP1‐WT, and pT3‐EF1α‐YAP1‐5SA were co‐injected into the tail vein. HTVi was performed in 5‐ to 6‐week‐old mice as previously described. Five to ten months after HTVi, mice were euthanized, and the liver tissues were collected for subsequent analysis.

### Tumor Xenograft Experiments

Male BALB/c nude mice (4 weeks old) were purchased from Jihui Laboratory Animal Care (Shanghai, China). SNU398 shNC or shTRIM65 cells (1 × 10^7^) were injected subcutaneously into the rear of the axilla of 5‐week‐old nude mice. The lengths and widths of the subcutaneous tumors were measured every seven days. Tumor size was calculated using the following formula: volume = (length × width^2^) / 2. After 28 days, the mice were sacrificed and the tumors were removed, weighed and photographed.

### RNA Extraction and Real Time Quantitative PCR (RT‐qPCR)

TRIzol reagent (Invitrogen, Cat#15596018CN) was used to extract total RNA from tissues and cell lysates. The abundances of target mRNAs were detected using a PrimeScript RT reagent kit (TaKaRa, Cat#RR037A) and TB Green Premix Ex Taq II (TaKaRa, Cat#RR820A) according to the manufacturer's protocols. The 2^−ΔΔCT^ method was used for relative quantitation. 18S rRNA were used as internal controls. Primers used in this study are listed in Table S4 (Supporting Information).

### RNA‐seq and Proteomics

RNA‐seq and proteomics were conducted by Genechem (Shanghai, China).

For RNA‐seq, total RNAs from shNC cells and shOGT cells were extracted. Then RNA‐seq libraries were constructed. Transcriptome sequencing was performed using Illumina Hiseq. SeqPrep and Sickle software were used for raw data quality control. Reference genome comparison was performed using TopHat software to obtain the expression value of each gene. Finally, Cuffdiff software was utilized for differential analysis.

The iTRAQ quantitative proteomics analysis was performed using a high‐resolution mass spectrometer, Q Exactive. The raw data were processed using Proteome Discoverer 2.1 software, and MASCOT 2.5 server was utilized to screen the data according to the criterion of FDR < 0.01, and to obtain highly reliable qualitative results. Differential proteins between groups were analyzed using the t test method.

### Cell Proliferation, Apoptosis and Cell Cycle Assay

For cell proliferation assay, 1000 cells per well were seeded into a 96‐well plate or 12‐well plate. For Cell counting kit‐8 (CCK‐8) assay, working solution was a mixture of 10 µL of CCK‐8 reagent (Beyotime Biotechnology, Cat#C0039) and 90 µL of fresh medium. After incubation at 37 °C for 2 h, the absorbance at 450 nm was detected using a SpectraMax iD5 Multi‐Mode Microplate Reader (Molecular Devices). For colony formation assay, after 7–10 days culture in the 12‐well plate, cell colonies were fixed with 4% paraformaldehyde, stained with 0.1% crystal violet and the number of colonies were calculated with ImageJ software.

Dead Cell Apoptosis Kit with Annexin V FITC and propidium iodide (PI, Invitrogen, Cat#V13242) was used for apoptosis analyses and PI/RNase staining buffer (BD Pharmingen, Cat#550 825) was used for cell cycle analyses according to the manufacturer's instruction.

### Ubiquitome Analysis

The ubiquitome analysis was conducted by Jingjie PTM Biolab Co.Inc. (Hangzhou, China). To enrich Kub modified peptides, tryptic peptides dissolved in NETN buffer (100 mm NaCl, 1 mm EDTA, 50 mm Tris‐HCl, 0.5% NP‐40, pH 8.0) were incubated with pre‐washed antibody beads (Cat#PTM‐1104, PTM Bio) at 4 °C overnight with shaking. The bound peptides were eluted from the beads with 0.1% trifluoroacetic acid. Finally, the eluted fractions were combined and vacuum‐dried. For LC‐MS/MS analysis, the resulting peptides were desalted with C18 ZipTips (Millipore) according to the manufacturer's instructions. The resulting MS/MS data were processed using MaxQuant search engine (v.1.6.15.0). Tandem mass spectra were searched against the human SwissProt database (20 395 entries) concatenated with reverse decoy database.

### Co‐Immunoprecipitation (Co‐IP) and Western Blot (WB) Assay

Cells were lysed with the lysis buffer (20 mm Tris‐HCl pH 8.0, 0.5% NP‐40, 200 mm NaCl, 1 mm EDTA) containing protease and phosphatase inhibitors (Sangon Biotech, Shanghai, China) for 30 min on ice. Then, protein was mixed with Protein A/G PLUS‐Agarose (Santa Cruz Biotechnology, Cat#sc‐2003) conjugated with indicated antibodies and rocked at 4 °C overnight. Beads were washed with lysis buffer and separated immunoprecipitated protein complex via boiling, followed by WB analysis, which was conducted conventionally. The primary antibodies used in this study were diluted 1:1000 in Universal Antibody Diluent® (NCM Biotech, Cat# WB500D) (except for GAPDH 1:10 000). The secondary antibodies used were IRDye® 800CW goat anti‐mouse/rabbit IgG secondary antibodies (LI‐COR Biosciences, Cat#926‐32210, Cat#926‐32211, 1:2000) and anti‐mouse/rabbit horseradish peroxidase‐linked antibodies (Cell Signaling Technology, Cat#7076S, Cat#7074S, 1:2000). Membranes were visualized with an Odyssey imaging system (LI‐COR Biosciences) or Amersham Imager 600 (GE Healthcare Life Sciences). The integrated density of the bands was analyzed with ImageJ software. Antibodies used are listed in Table S5 (Supporting Information).

### Untargeted Metabolomics

The untargeted metabolomics profiling was performed on XploreMET platform (Metabo‐Profile, Shanghai, China). The sample preparation procedures were referred in their previously published methods.^[^ ^40^ ^]^ The raw data generated by GC‐TOF/MS were processed using XploreMET (v3.0, Metabo‐Profile, Shanghai, China). The metabolomics data had been deposited to the MetaboLights (MTBLS8709).

### UDP‐GlcNAc Measurement

The concentration of UDP‐GlcNAc in liver tissues or cell lines was detected by UDP‐GlcNAc ELISA kit (Abmart, Cat#AB‐15785) according to the manufacturer's protocol. Remove the particulates of tissue homogenates or cell lysates by centrifugation. After incubation, the stop solution was measured at 450 nm using a spectrophotometer (Molecular Devices) and the concentration of UDP‐GlcNAc in the samples was calculated based on the calibration standard curve.

### Mass spectrometry Analysis (MS) of Uridine, Uracil, and UMPS

MS of uridine, uracil and UMPS was conducted by Metabo‐Profile Biotechnology Corporation (Shanghai, China) with the UPLC‐MS/MS system (ACQUITY UPLC‐Xeco TQ‐S, Milford). Briefly, phases A and B were 50% and 80% ammonium acetate (15 mm), respectively. The mass spectrometer was operated as follows: capillary voltage, 3 kV; source temperature, 150 °C; de‐solvent temperature, 500 °C; de‐solvent airflow, 1000L Hr^−1^. Raw data was analyzed by MassLynx software v4.1 (Milford) to calibrate and quantify the peak of uridine, uracil and UMPS. The iMAP software v1.0 (Metabo‐Profile, Shanghai, China) was applied for statistics.

### Chromatin Immunoprecipitation (ChIP) ‐qPCR Assay

Cells (4 × 10^6^) were harvested and treated with 1% formaldehyde for 10 min at room temperature to cross‐link proteins to DNA. Glycine solution was added to stop the cross‐linking process. ChIP assays were performed using the Pierce Magnetic ChIP Kit (Thermo Fisher Scientific, Cat#26 157) according to the manufacturer's manuals. Purified DNA was subjected to qPCR using TB Green™ Premix Ex Taq™ II (TaKaRa, Cat#RR820A). Antibodies and primers used in ChIP‐qPCR were anti‐YAP1 (Proteintech, Cat#13584‐1‐AP, 5 µL sample^−1^) and anti‐CREB (Cell Signaling Technology, Cat#9197, 5 µL sample^−1^).

### Immunofluorescence (IF)

IF assays were performed as described everywhere. Cells were fixed with 4% paraformaldehyde (Biosharp, Cat#BL539A) for 15 min at room temperature, permeabilized with 0.5% TritonX‐100 for 20 min at 37 °C, blocked with 5% bovine serum albumin in PBS for 30 min at room temperature, followed by incubating with the primary antibodies at 4 °C overnight. After washing, cells were incubated with corresponding secondary antibodies at 37 °C for 30 min. Subsequently, 4′,6‐ diamidino‐2‐phenylindole (DAPI, Sigma, Cat#D8417) was applied to stain the nuclei. The images were captured using the confocal laser scanning microscope (Carl Zeiss) with identical exposure settings in each experiment replicate. Antibodies used are listed in Table S5 (Supporting Information).

### Immunohistochemistry (IHC)

The liver tissues were fixed in 4% paraformaldehyde (Biosharp, Cat#BL539A) overnight. Tissues were embedded in paraffin. Then, the slides were deparaffinated, hydrated, heated for antigen retrieved, blocked of nonspecific staining, and then incubated with primary antibody at 4 °C overnight. After the incubation with SignalStain® Boost IHC Detection Reagent (Cell Signaling Technology, Cat# 8114) for 1 h at room temperature, the diaminobenzidine chromogenic substrate was applied for 10 min at room temperature to visualization. The panoramic scanning was conducted by Runnerbio Corporation (Shanghai, China). The staining intensity analysis was interpreted by two pathologists who were blinded to the information of specimens. Integrated optical density (IOD) was analyzed via Image Pro Plus software.

### Protein Stability Assay

The stability of TRIM65 and NF2 protein was assessed as previously reported with slight modification.^[^ ^10^ ^]^ Briefly, cells were treated with 100 µg ml^−1^ CHX (Sigma‐Aldrich, Cat#239 764) to arrest translation and then collected at 0, 2, 4, 8, 16, and 30 h, respectively. Protein was extracted from the cells and analyzed by WB.

### Nuclear and Cytoplasmic Extraction Assay

Nuclear and cytoplasmic extracts were obtained with the NE‐PER Nuclear and Cytoplasmic Extraction Reagents (Thermo Fisher Scientific, Cat#78 833) according to the manufacturer's instructions. Afterward, proteins derived from nucleolus and cytoplasm were evaluated by WB.

### Dual‐Luciferase Reporter Assay

The promoter sequence of UMPS, CTGF, and FASN was cloned into the upstream of luciferase gene in the pGL4.21 vector. HEK‐293T cells were priorly seeded in 24‐well plates followed by co‐transfection with constructed reporter plasmids or empty vectors. Afterward, cells were lysed in lysis buffer and the luciferase activity was examined using the Dual‐Luciferase® Reporter Assay System (Promega, Cat#E1910) according to the manufacturer's instructions.

### Plasma Liver Function Test

The peripheral whole blood was collected and anticoagulated with heparin sodium (Biosharp, Cat#BS145). Plasma was acquired from the supernatant after centrifuging in the speed of 3000 rpm at 4 °C for 5 min. Subsequently, the level of Alanine aminotransferase (ALT) and Aspartate Transaminase (AST) were assessed using the Fully Automated Biochemical Analyzer (Abbott) as the manufacturer's instructions.

### BODIPY 493/503 Staining

For lipid droplets examination, BODIPY 493/503 (MCE, Cat# HY‐W090090) working solution was prepared in PBS. Cells were cultured on sterile coverslips and tissue section was obtained priorly. Then 2 and 50 µm working solution were added to completely cover the cells and tissues, respectively, followed by the incubation for 30 min at 37 °C away from light. Afterward, cells and tissues were washed and observed using confocal laser scanning microscope (Carl Zeiss).

### Free Fatty Acids Assay

Free fatty acids were examined using the Non‐esterified Free Fatty Acids (NEFA/FFA) Colorimetric Assay Kit (Elabscience, Cat# E‐BC‐K013‐M) as the protocol. Briefly, extracting solution was added to fresh tissues and homogenized at 4 °C for 2 h. Then lysates were centrifuged for 10 min at 10 000 g. The triplicate supernatants, control solution and diluted standards were transferred to new tubes respectively. Afterward, reaction solution was added to standards and tests. All tubes were vortexed fully for 3 min followed by standing for 3 min at room temperature. The supernatant was subjected to detect the OD value using spectrophotometer (Molecular Devices) at 715 nm.

### Macrophage Induction and Co‐Culturing

THP‐1 cells were stimulated using 200 nm phorbol 12‐myristate 13‐acetate (PMA, MCE, Cat# HY‐18739) for 3 days. Then PMA‐containing media was removed, and differentiated cells were incubated in fresh media. At the same time, HCC cells with indicated treatment were seeded in chambers (Jet Biofil, Guangzhou, China) priorly and co‐cultured with the PMA induced THP‐1 cells for further 3 days. Afterward, the phenotype of the macrophage polarization was evaluated by RT‐qPCR.

### Flow Cytometric Measurements for Immunophenotype in Liver

Fresh mice liver tissues were cut up sufficiently and suspended in DMEM containing 160 U mL^−1^ collagenase IV (Gibco, Cat# 17 104 019), 2U mL^−1^ DNase (Sigma‐Aldrich, Cat# 4 716 728 001), 1% PS and 2.5% FBS on ice. The obtained tissue suspension was fully mixed and incubated in 37 °C for 30 min. Then 10% FBS was added to terminate the digestion. The whole mixture was transferred to 100 µm cell strainers and centrifuged for 3 min at 150 g, 4 °C. The pellets were resuspended in red blood cell lysis buffer (Beyotime, China) and washed with PBS. After that, the pellets were stained in stain buffer (BD Pharmingen) containing specific anti‐mouse antibodies and incubated for 30 min at 4 °C away from light. The data were acquired on BD Fortessa flow cytometer and analyzed using TreeStar FlowJo software. Antibodies used in this assay are listed in Table S6 (Supporting Information) and were diluted 1:200 in Stain buffer (BD Pharmingen, Cat#554 657).

### Statistics

All experiments were conducted in triplicate. For quantification of the nuclear/cytoplasmic fluorescence ratio of YAP1, 10–20 cells in 3–5 representative images were quantified with Image J for each sample. Statistical tests were performed using GraphPad Prism 8.0 with Student's two‐tailed unpaired t test for pairwise comparisons, one‐way analysis of variance (ANOVA) for multiple comparisons, two‐way ANOVA for multiple comparisons involving two independent variables. Pearson's Correlation analysis was used for correlation coefficient. Survival curves were evaluated by Kaplan–Meier analysis (log‐rank test). The association between TRIM65 expression and prognosis of HCC patients was appraised using a cox proportional hazards regression model. Data are presented as mean ± SD. A *p* < 0.05 was considered significant. Statistical significance was defined as *** (*p* < 0.001), ** (*p* < 0.01), * (*p* < 0.05), NS = not significant.

### Ethical Statement

Tissues were obtained from Shanghai Tenth People's Hospital with the approval of the ethics committee. All the patient tissues were obtained with the written consent of each participant. All procedures related to animal studies were performed in accordance with the guidelines approved by the Animal Research Ethics Committee of Shanghai Tenth People's Hospital.

## Conflict of Interest

The authors declare no conflict of interest.

## Author Contributions

Z.B., C.X., and X.W. contributed equally to this work. S.F. performed conceptualization, supervision, funding acquisition, wrote, reviewed, and edited the draft, and provided resources. P.Q. performed conceptualization, supervision, funding acquisition, and provided resources. W.X. performed conceptualization, supervision, wrote, reviewed, and edited the draft, and provided resources. B.Z. and X.C. performed methodology, investigation, and wrote the original draft. Z.B., X.Y., L.L., Z.S., H.N., Y.F., Z.Y., and X.S. performed investigation.

## Supporting information

Supporting Information

Supplemental Table 1

Supplemental Table 2

## Data Availability

The data that support the findings of this study are openly available in MetaboLights at https://www.ebi.ac.uk/metabolights, reference number 8709.
