# Supplementary material for: TRIM65/NF2/YAP1 Signaling Coordinately Orchestrates Metabolic and Immune Advantages in Hepatocellular Carcinoma
Source: Adv Sci (Weinh). 2024 Jul 15;11(35):2402578. doi: 10.1002/advs.202402578 (PMC11425264; doi:10.1002/advs.202402578)
Supplement: Supplementary file 1 — Supporting Information [file ADVS-11-2402578-s001.pdf]

Supporting information

TRIM65/NF2/YAP1 signaling coordinately orchestrates metabolic and immune advantages in hepatocellular carcinoma

Zhixuan Bian, Chang Xu, Xiaoying Wang, Baohua Zhang, Yixuan Xiao, Li Liu, Shasha Zhao, Nan Huang, Fengjiao Yang, Yue Zhang, Shaobo Xue, Xiongjun Wang, Qiuhui Pan*, Fenyong Sun**

This file includes:

Supplemental Figures S1-S7

Supplemental tables S3-S6

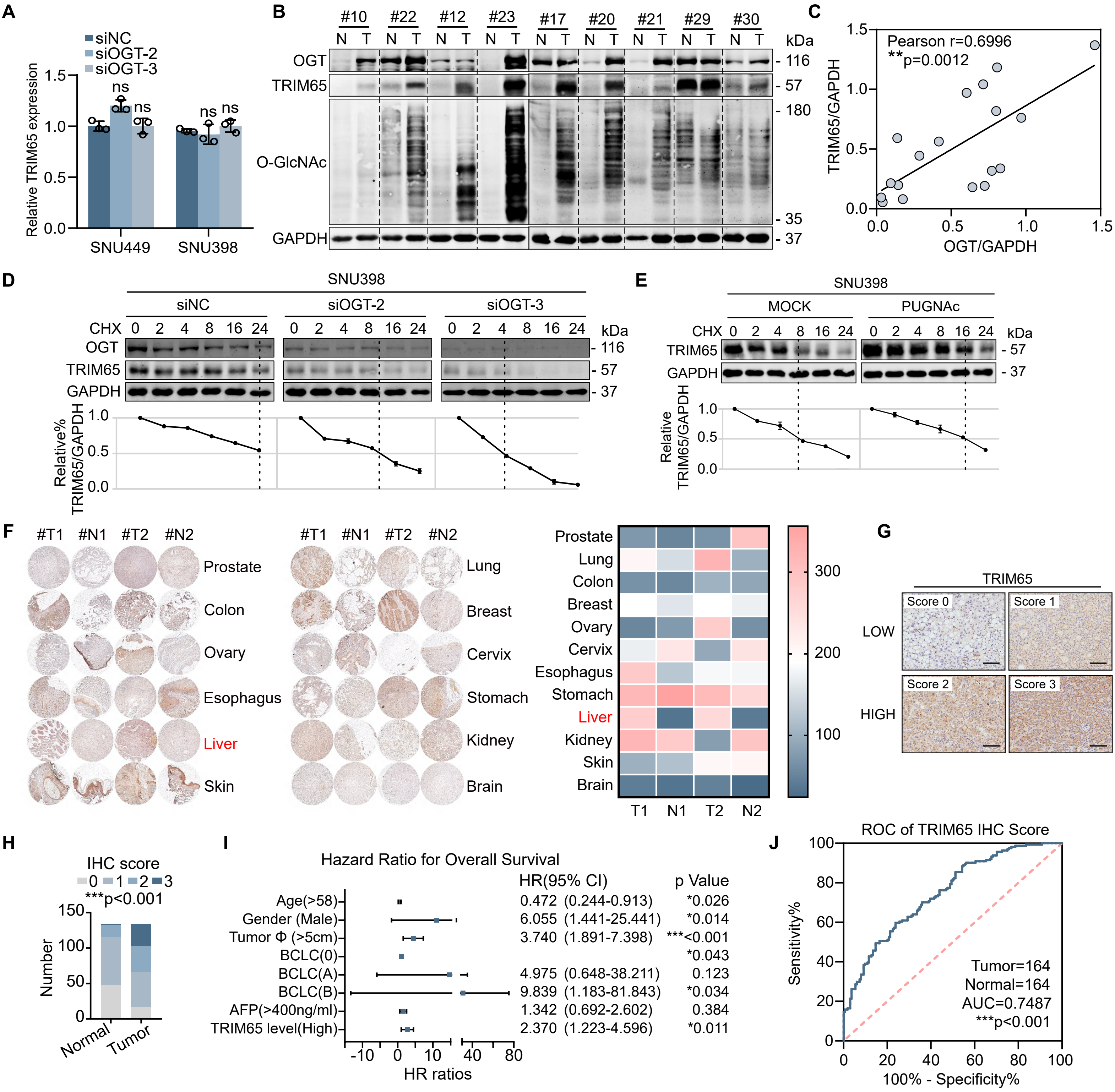

A
siNC
siOGT-2
siOGT-3
Relative TRIM65 expression
ns
SNU449
SNU398
B
#10 #22 #12 #23 #17 #20 #21 #29 #30
N T
kDa
OGT
TRIM65
O-GlcNAc
GAPDH
116
57
180
35
37
C
Pearson r=0.6996
**p=0.0012
TRIM65/GAPDH
OGT/GAPDH
D
SNU398
siNC
siOGT-2
siOGT-3
CHX 0 2 4 8 16 24
Relative% TRIM65/GAPDH
E
SNU398
MOCK
PUGNAc
Relative TRIM65/GAPDH
F
#T1 #N1 #T2 #N2
Prostate
Colon
Ovary
Esophagus
Liver
Skin
Lung
Breast
Cervix
Stomach
Kidney
Brain
T1 N1 T2 N2
300
200
100
G
TRIM65
LOW
HIGH
Score 0
Score 1
Score 2
Score 3
H
IHC score
0 1 2 3
***p<0.001
Number
Normal
Tumor
I
Hazard Ratio for Overall Survival
HR(95% CI)
p Value
Age(>58) 0.472 (0.244-0.913) *0.026
Gender (Male) 6.055 (1.441-25.441) *0.014
Tumor Φ (>5cm) 3.740 (1.891-7.398) ***<0.001
BCLC(0) *0.043
BCLC(A) 4.975 (0.648-38.211) 0.123
BCLC(B) 9.839 (1.183-81.843) *0.034
AFP(>400ng/ml) 1.342 (0.692-2.602) 0.384
TRIM65 level(High) 2.370 (1.223-4.596) *0.011
HR ratios
J
ROC of TRIM65 IHC Score
Sensitivity%
100% - Specificity%
Tumor=164
Normal=164
AUC=0.7487
***p<0.001

Figure S1 The pan-cancer analysis of TRIM65 expression and its clinical significance in HCC.

(A) RT-qPCR analysis of TRIM65 expression in OGT knockdown cells. (B)Western blot (WB) analysis of TRIM65, OGT and O-GlcNAc expression in paired HCC and adjacent normal tissues. (C) Correlation between OGT and TRIM65 expression based on the grey value. (D-E) Protein stability assay in SNU398 cells with indicated treatments. TRIM65 levels were normalized by GAPDH and the 0 h points were set to 100%. (F) Immunohistochemistry (IHC) staining of TRIM65 in multiple cancer tissue microarray. (G-H) IHC staining of TRIM65 in HCC tissue microarray (n=136). IHC scores were determined according to the intensity. (I) Forest plot depicting multivariate analysis of overall survival of HCC patients based on tissue microarray (Cox proportional hazards regression model). (J) ROC curve for tissue-derived TRIM65 in CRC diagnosis. *** ($p < 0.001$), ** ($p < 0.01$), * ($p < 0.05$). One-way ANOVA for multiple comparisons.

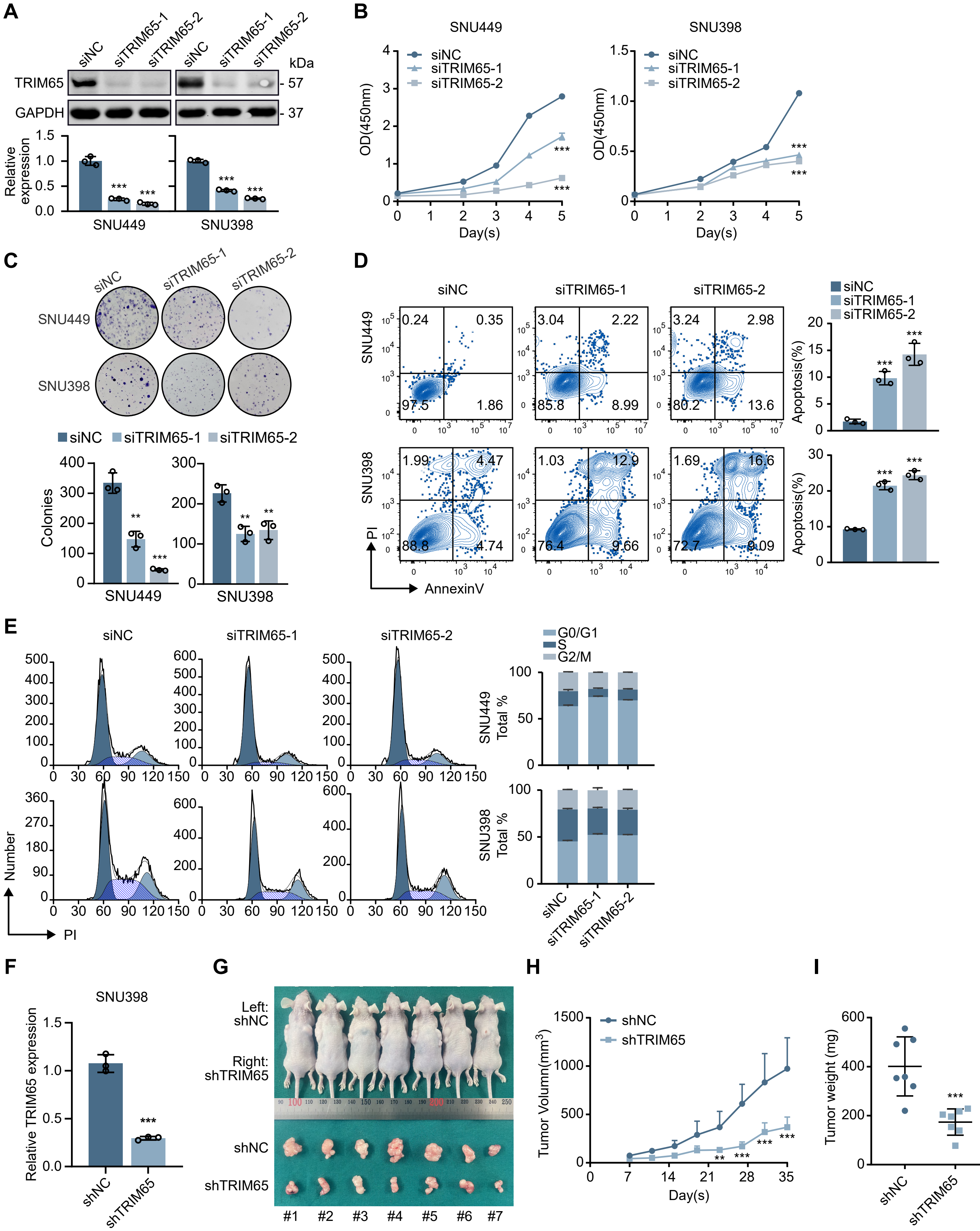
A
siNC siTRIM65-1 siTRIM65-2 siNC siTRIM65-1 siTRIM65-2
kDa
TRIM65 - 57
GAPDH - 37
Relative expression
SNU449 SNU398
B
SNU449
SNU398
siNC
siTRIM65-1
siTRIM65-2
OD(450nm)
Day(s)
C
SNU449
SNU398
Colonies
D
siNC siTRIM65-1 siTRIM65-2
Apoptosis(%)
PI
AnnexinV
E
G0/G1
S
G2/M
SNU449 Total %
SNU398 Total %
Number
PI
F
SNU398
Relative TRIM65 expression
shNC
shTRIM65
G
Left: shNC
Right: shTRIM65
shNC
shTRIM65
#1 #2 #3 #4 #5 #6 #7
H
shNC
shTRIM65
Tumor Volumn(mm3)
Day(s)
I
Tumor weight (mg)
shNC
shTRIM65

Figure S2 TRIM65 facilitates HCC progression *in vitro* and *in vivo*.
(A) The transfection efficiency of siRNAs against TRIM65 (siTRIM65) in SNU449 and SNU398 cells were examined by WB and RT-qPCR. (B-C) The proliferation of SNU449 and SNU398 cells transfected with siTRIM65 or negative control (siNC) were assessed by CCK8 (B) and colony formation assay (C). (D-E) Apoptosis (D) and cell cycle (E) analysis were measured by flow cytometry in HCC cells transfected with siNC or siTRIM65. (F) The efficiency of SNU398 cells with TRIM65 stably knockdown assessed by RT-qPCR. (G) Tumors dissected from seven nude mice subcutaneously injected with shNC (left) and shTRIM65 (right) SNU398 cells. (H) Tumor volumes were measured on indicated days and calculated for tumor growth curves. (I) Tumor weights of the dissected tumors. Data are presented as mean ± SD. *** ($p < 0.001$), ** ($p < 0.01$), *($p < 0.05$). Student's two-tailed unpaired t-test for pairwise comparisons and one-way ANOVA for multiple comparisons.

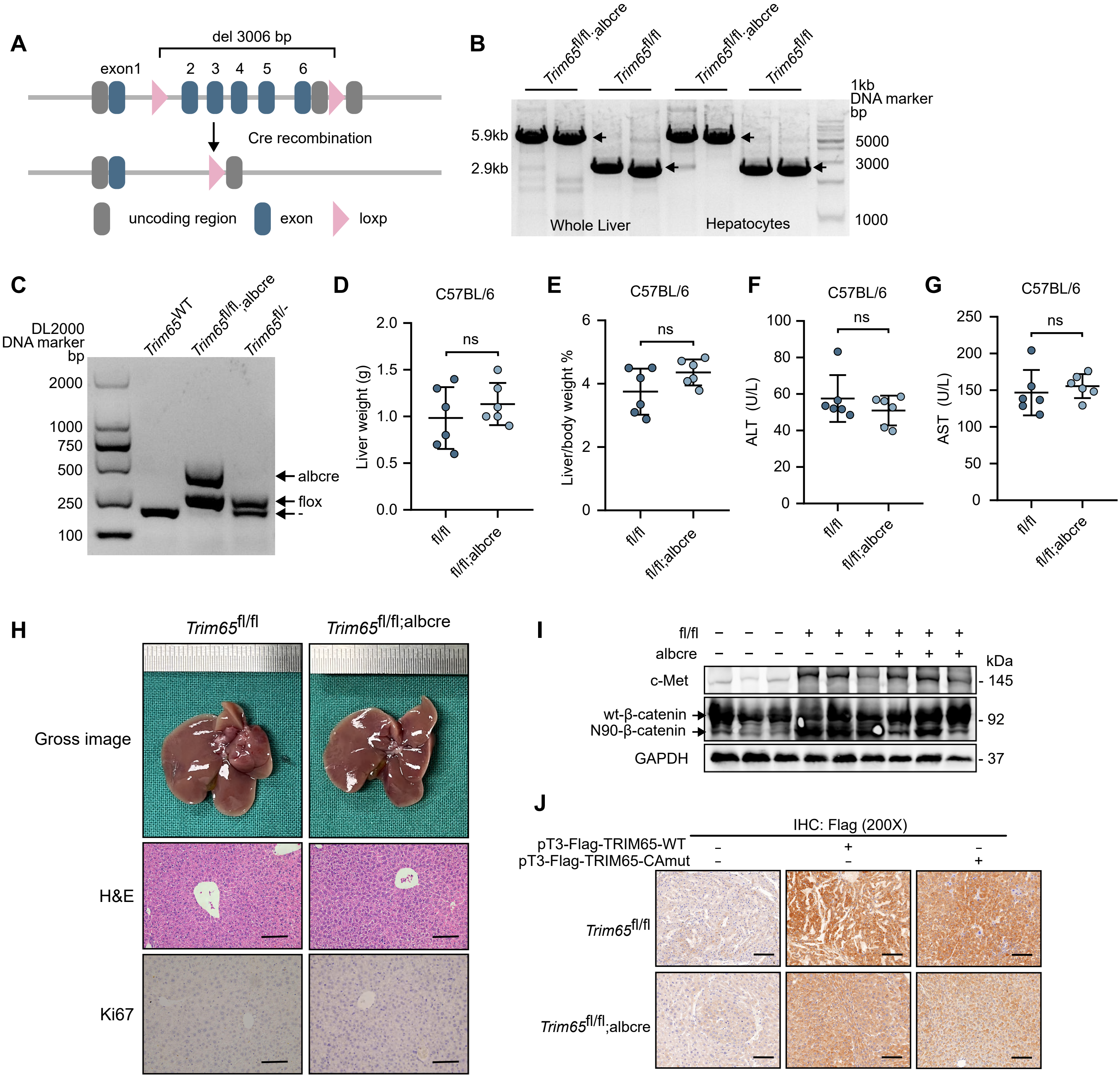
A
del 3006 bp
exon1
2 3 4 5 6
Cre recombination
uncoding region
exon
loxp
B
Trim65fl/fl,albcre
Trim65fl/fl
Trim65fl/fl,albcre
Trim65fl/fl
1kb DNA marker bp
5.9kb
2.9kb
5000
3000
1000
Whole Liver
Hepatocytes
C
DL2000 DNA marker bp
Trim65WT
Trim65fl/fl,albcre
Trim65fl/-
2000
1000
750
500
250
100
albcre
flox
-
D
C57BL/6
Liver weight (g)
ns
fl/fl
fl/fl;albcre
E
C57BL/6
Liver/body weight %
ns
F
C57BL/6
ALT (U/L)
ns
G
C57BL/6
AST (U/L)
ns
H
Trim65fl/fl
Trim65fl/fl;albcre
Gross image
H&E
Ki67
I
fl/fl
albcre
kDa
c-Met
wt-β-catenin
N90-β-catenin
GAPDH
145
92
37
J
IHC: Flag (200X)
pT3-Flag-TRIM65-WT
pT3-Flag-TRIM65-CAmut
Trim65fl/fl
Trim65fl/fl;albcre

Figure S3 Genotype identification and characteristics of liver specific TRIM65 knockout mice.
(A) Schematic diagram of the Cre/loxp conditional knockout system. (B) PCR using DNA from the whole liver or hepatocytes of *Trim65*$^{fl/fl}$;Alb-Cre and *Trim65*$^{fl/fl}$ mice. Products were visualized by electrophoresis. (C) Multiple PCR of the genome DNA from *Trim65*^{WT}, *Trim65*$^{fl/fl}$;Alb-Cre and *Trim65*$^{fl/fl}$ mice. Products were visualized by electrophoresis. (D-E) Liver weight (D) and liver to body weight ratio (E) of *Trim65*$^{fl/fl}$;Alb-Cre and *Trim65*$^{fl/fl}$ mice livers. (F-G) The ALT (F) and AST (G) level in *Trim65*$^{fl/fl}$;Alb-Cre and *Trim65*$^{fl/fl}$ mice plasma. (H) Representative gross image of livers, H&E and Ki67 staining in *Trim65*$^{fl/fl}$;Alb-Cre and *Trim65*$^{fl/fl}$ mice livers. Scale bar=100μm. (I) WB analysis of c-Met and β-catenin in *Trim65*$^{fl/fl}$;Alb-Cre and *Trim65*$^{fl/fl}$ mice liver tissues from the MET/N90 HCC model. (J) IHC staining of Flag-TRIM65 in *Trim65*$^{fl/fl}$;Alb-Cre and *Trim65*$^{fl/fl}$ mice liver tissues with MET/N90, MET/N90/TRIM65-WT or MET/N90/TRIM65-CAmut co-injection respectively. Data are presented as mean ± SD. *** ($p < 0.001$), ** ($p < 0.01$), * ($p < 0.05$). Student's two-tailed unpaired t-test for pairwise comparisons.

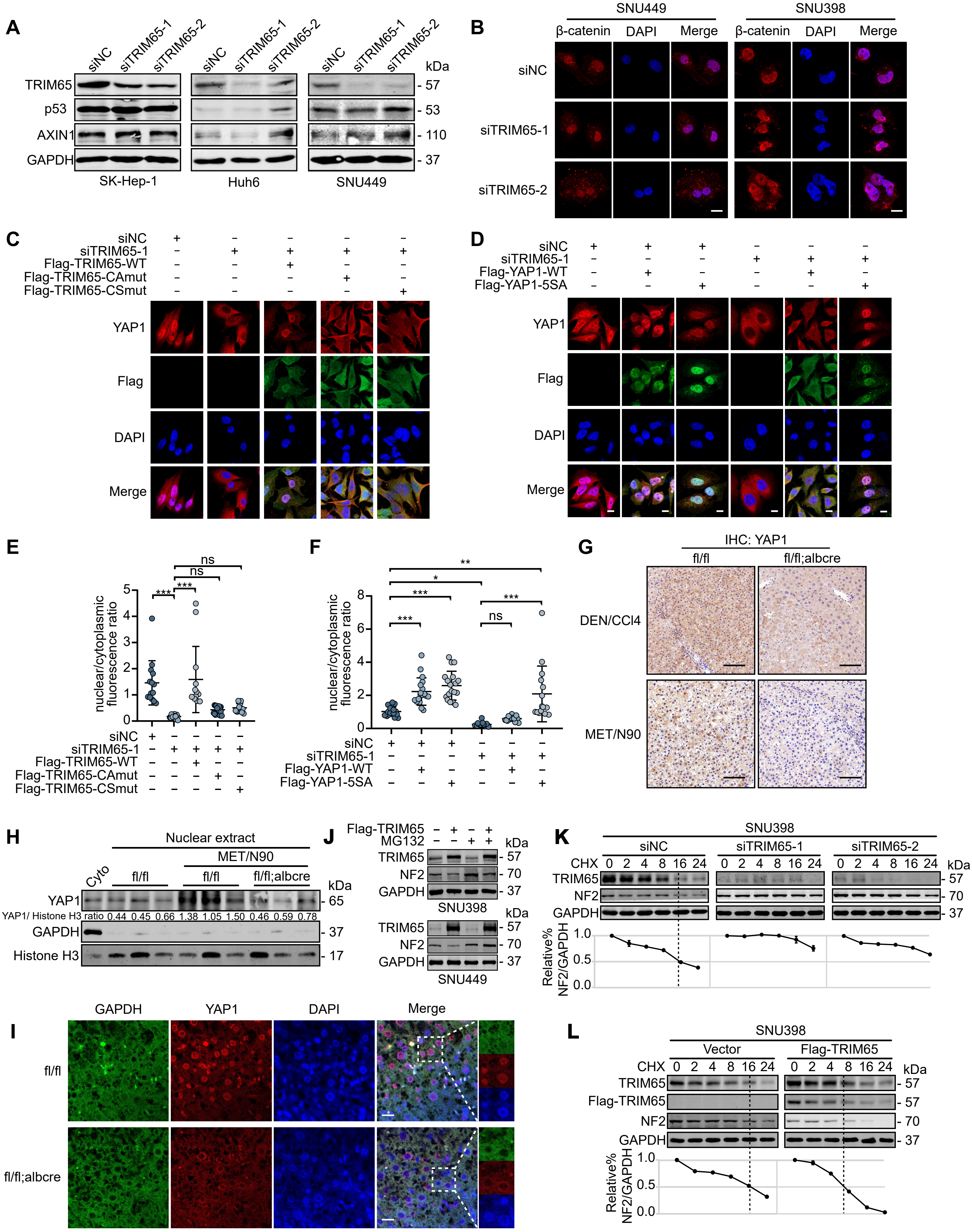

A
siNC
siTRIM65-1
siTRIM65-2
kDa
TRIM65
p53
AXIN1
GAPDH
SK-Hep-1
Huh6
SNU449
B
SNU449
SNU398
β-catenin
DAPI
Merge
siNC
siTRIM65-1
siTRIM65-2
C
Flag-TRIM65-WT
Flag-TRIM65-CAmut
Flag-TRIM65-CSmut
YAP1
Flag
DAPI
Merge
D
Flag-YAP1-WT
Flag-YAP1-5SA
E
F
nuclear/cytoplasmic fluorescence ratio
ns
***
*
**
G
IHC: YAP1
fl/fl
fl/fl;albcre
DEN/CCl4
MET/N90
H
Nuclear extract
Cyto
YAP1/ Histone H3 ratio 0.44 0.45 0.66 1.38 1.05 1.50 0.46 0.59 0.78
Histone H3
J
Flag-TRIM65
MG132
NF2
SNU398
SNU449
K
CHX 0 2 4 8 16 24
Relative% NF2/GAPDH
I
L
Vector

Figure S4 TRIM65 promotes the nuclear import of YAP1 through regulating the stability of NF2 protein.

(A) Expression of TRIM65, p53 and AXIN1 in p53 wild type liver cancer cells (SK-Hep-1 and Huh6) and SNU449 transfected with siNC or si*TRIM65*. (B) The localization of β-catenin was detected by immunofluorescence (IF) in SNU449 and SNU398 cells transfected with siNC or si*TRIM65*. Scale bar=10μm. (C) The localization of exogeneous Flag-TRIM65 (green) and YAP1 (red) were detected by IF using anti-Flag and anti-YAP1 antibodies in SNU398 cells with indicated treatments. Nuclei were stained with DAPI (blue). Scale bar=10μm. (D) The localization of exogeneous Flag-YAP1 (green) and total YAP1 (red) were detected by IF using anti-Flag and anti-YAP1 antibodies in SNU398 cells co-transfected with si*TRIM65* and *YAP1* overexpressed plasmids (wild type or activating mutation). Scale bar=10μm. (E-F) The statistics of the YAP1 nuclear and cytoplasmic fluorescence ratio upon corresponding IF signals. (G) IHC staining of YAP1 in *Trim65*$^{fl/fl}$;Alb-Cre and *Trim65*$^{fl/fl}$ mice liver tissues under DEN/CCl4 or MET/N90 treatment. Scale bar=100μm. (H) YAP1 expression in nuclear and cytoplasmic extractions from MET/N90 mice liver tissues were analyzed by WB. (I) Tissue IF assays of MET/N90 mice livers were conducted to verify the YAP1 localization. Scale bar=100μm. (J) NF2 expression in cells treated with MG132 (10μM) was analyzed by WB. (K-L) Protein stability assay of NF2 in SNU398 cells with TRIM65-knockdown or TRIM65-overexpression was measured by WB. The levels of NF2 were normalized by GAPDH. Data are presented as mean ± SD. *** ($p < 0.001$), ** ($p < 0.01$), * ($p < 0.05$). One-way ANOVA for multiple comparisons.

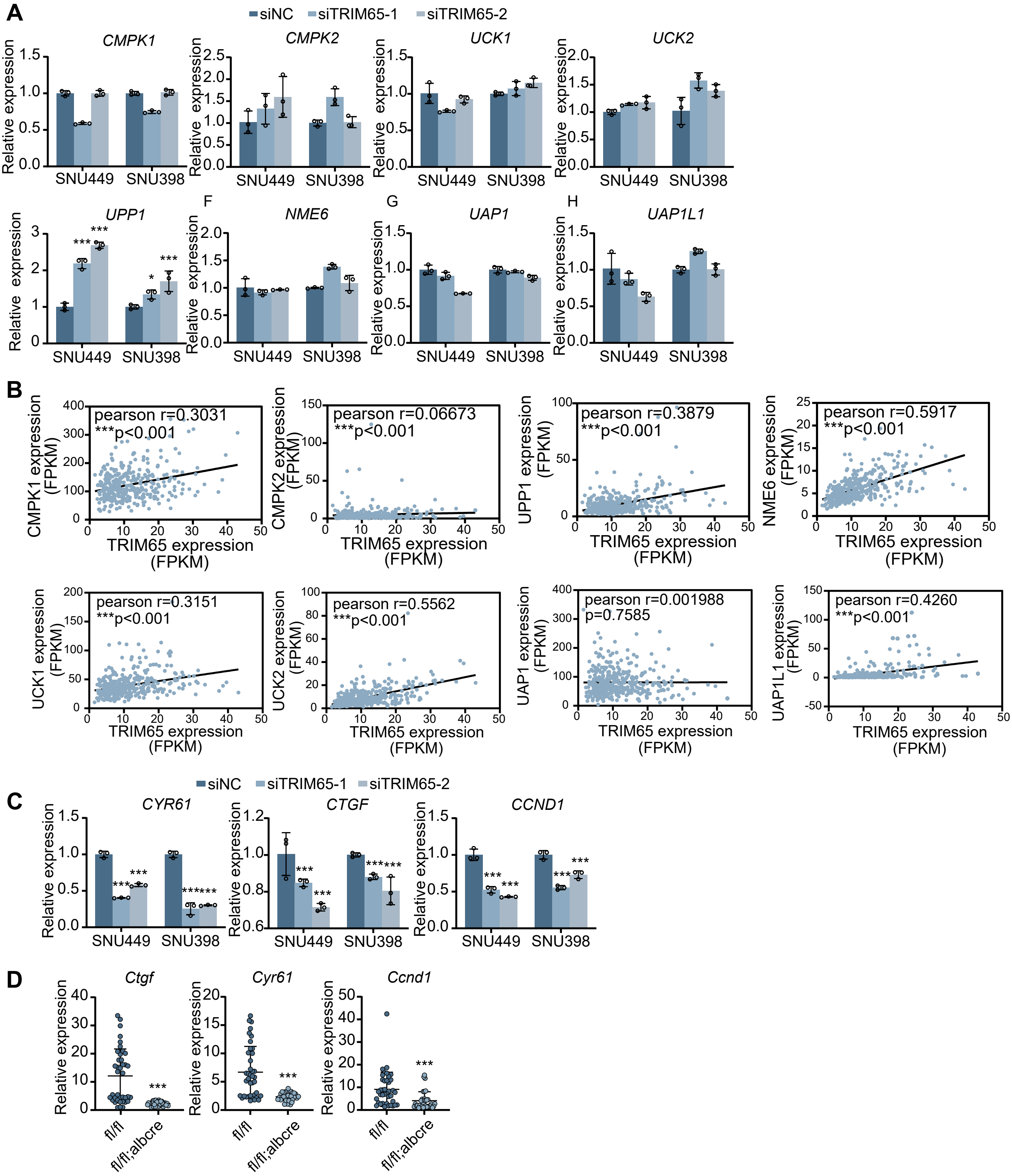
A
siNC siTRIM65-1 siTRIM65-2
CMPK1
CMPK2
UCK1
UCK2
UPP1
F
NME6
G
UAP1
H
UAP1L1
Relative expression
SNU449 SNU398
B
pearson r=0.3031
***p<0.001
pearson r=0.06673
***p<0.001
pearson r=0.3879
***p<0.001
pearson r=0.5917
***p<0.001
pearson r=0.3151
***p<0.001
pearson r=0.5562
***p<0.001
pearson r=0.001988
p=0.7585
pearson r=0.4260
***p<0.001
CMPK1 expression (FPKM)
CMPK2 expression (FPKM)
UPP1 expression (FPKM)
NME6 expression (FPKM)
UCK1 expression (FPKM)
UCK2 expression (FPKM)
UAP1 expression (FPKM)
UAP1L1 expression (FPKM)
TRIM65 expression (FPKM)
C
siNC siTRIM65-1 siTRIM65-2
CYR61
CTGF
CCND1
D
Ctgf
Cyr61
Ccnd1
fl/fl
fl/fl;albcre

Figure S5 The positive feedback loop to accelerate HCC progression composed of TRIM65, uracil metabolism and O-GlcNAcylation.

(A) Relative expression of enzymes relevant to uracil metabolism including *CMPK1*, *CMPK2*, *UCK1*, *UCK2*, *UPP1*, *NME6*, *UAP1* and *UAP1L1* were detected by RT-qPCR in SNU449 and SNU398 cells with TRIM65-silencing. (B) The correlation analysis using HCC datasets (n=374) from TCGA database between *TRIM65* and ten enzymes in uracil metabolism. Pearson correlation coefficients were calculated respectively. (C) Relative mRNA expression of *CYR61*, *CTGF* and *CCND1* were detected by RT-qPCR in SNU449 and SNU398 cells with *TRIM65*-knockdown. (D) Relative mRNA expression of *Ctgf*, *Cyr61* and *Ccnd1* were measured by RT-qPCR in *Trim65*$^{fl/fl}$;Alb-Cre and *Trim65*$^{fl/fl}$ mice liver tissues. Data are presented as mean ± SD. *** ($p < 0.001$), ** ($p < 0.01$), * ($p < 0.05$). Student's two-tailed unpaired t-test for pairwise comparisons and one-way ANOVA for multiple comparisons.

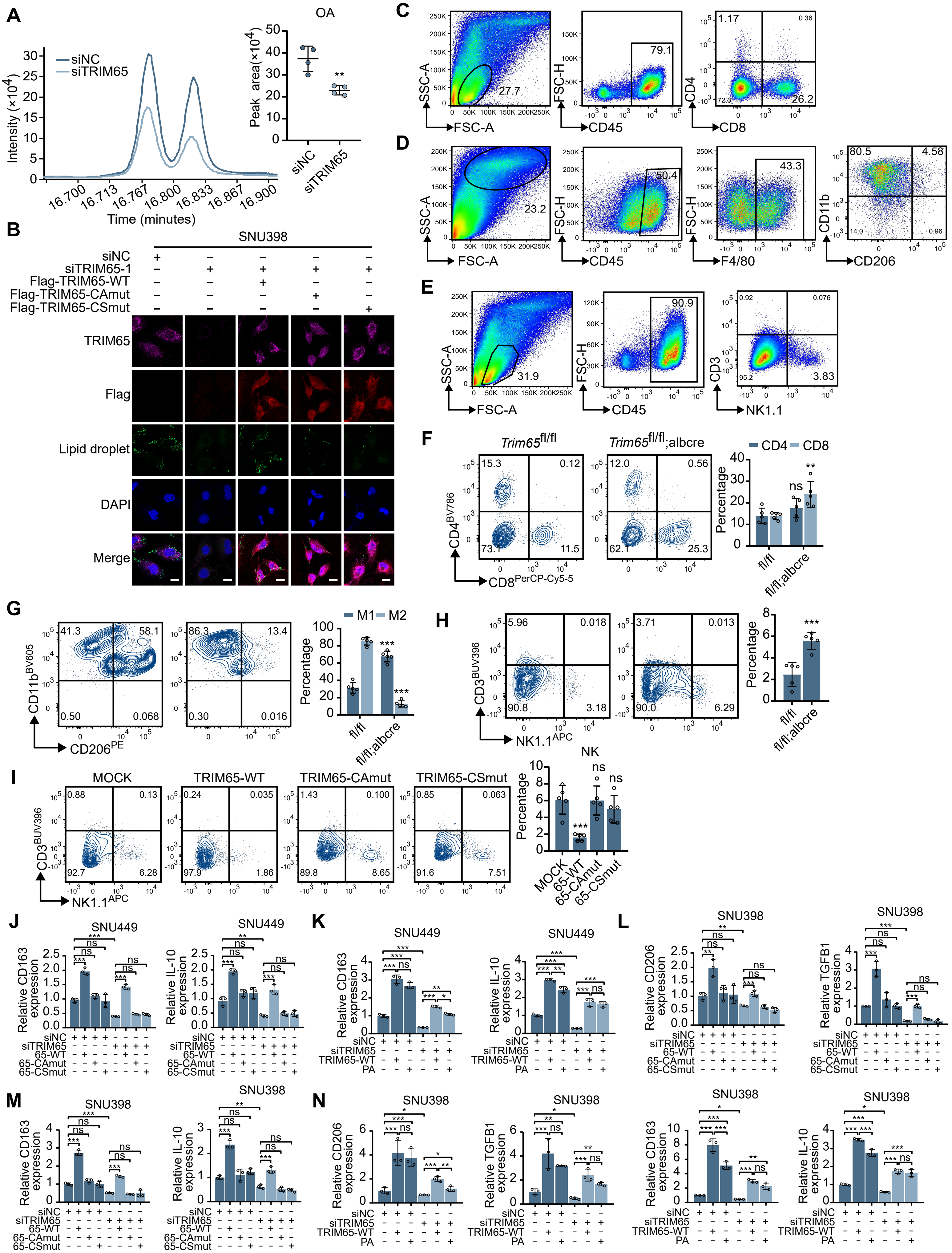
A
OA
siNC
siTRIM65
Intensity (×10^4)
Time (minutes)
Peak area (×10^4)
B
SNU398
siNC
siTRIM65-1
Flag-TRIM65-WT
Flag-TRIM65-CAmut
Flag-TRIM65-CSmut
TRIM65
Flag
Lipid droplet
DAPI
Merge
C
SSC-A
FSC-A
FSC-H
CD45
CD4
CD8
D
F4/80
CD11b
CD206
E
CD3
NK1.1
F
Trim65fl/fl
Trim65fl/fl;albcre
CD4BV786
CD8PerCP-Cy5-5
CD4
CD8
Percentage
fl/fl
fl/fl;albcre
G
M1
M2
CD11bBV605
CD206PE
H
CD3BUV396
NK1.1APC
I
MOCK
TRIM65-WT
TRIM65-CAmut
TRIM65-CSmut
NK
65-WT
65-CAmut
65-CSmut
J
SNU449
Relative CD163 expression
Relative IL-10 expression
K
TRIM65-WT
PA
L
SNU398
Relative CD206 expression
Relative TGFB1 expression
M
N

Figure S6 TRIM65 contributed to the accumulation of palmitic acid, thereby stimulating the immunosuppressive tumor microenvironment of HCC.

(A) Mass spectrometry analysis of Oleic acid (OA) in SNU449 cells transfected with siNC or si*TRIM65*. (B) TRIM65, Flag and lipid droplets were visualized by IF assay in SNU398 cells transfected with indicated treatments. DAPI was used to stain nuclei. Scale bar=10μm. (C-E) The gating strategies of the flow cytometry in the detection of T cells (C), macrophages (D), and NK cells (E). (F-H) T cells (F), macrophages (G) and NK cells (H) from *Trim65*$^{fl/fl}$ and *Trim65*$^{fl/fl}$;Alb-Cre mice liver tissues were detected using flow cytometry in MET/N90 HCC model. (I) The NK cells from mice liver tissues were detected using flow cytometry after liver in-situ injection with Hepa1-6 cells, which were transfected with empty vector, *TRIM65*-WT, CAmut and CSmut, respectively. (J-N) Relative expression of *CD163*, *IL-10*, *CD206* and *TGFB1* in THP-1 cells detected by RT-qPCR. (J, L-M) THP-1 cells were co-cultured with SNU449 and SNU398 cells transfected with siNC, si*TRIM65*, or together with *TRIM65*-WT, CAmut or CSmut respectively. (K, N) THP-1 cells were co-cultured with SNU449 and SNU398 cells transfected with siNC, si*TRIM65*, or together with *TRIM65*-WT or PA treatment respectively. Data are presented as mean ± SD. *** ($p < 0.001$), ** ($p < 0.01$), * ($p < 0.05$). Student's two-tailed unpaired t-test for pairwise comparisons and one-way ANOVA for multiple comparisons.

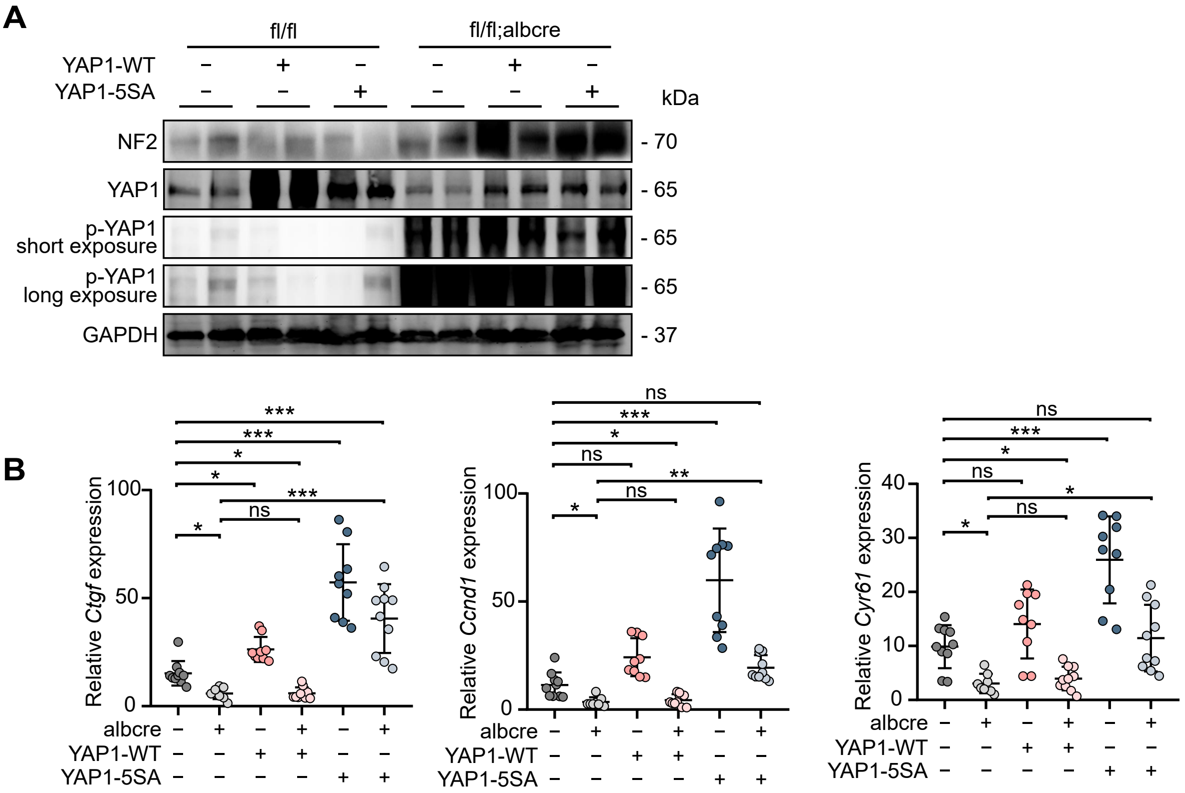
A
fl/fl
fl/fl;albcre
YAP1-WT
YAP1-5SA
kDa
NF2
YAP1
p-YAP1
short exposure
p-YAP1
long exposure
GAPDH
- 70
- 65
- 65
- 65
- 37
B
Relative Ctgf expression
Relative Ccnd1 expression
Relative Cyr61 expression
albcre
YAP1-WT
YAP1-5SA
ns
*
**
***

Figure S7. The efficiency of YAP1 retrieved and the expression of YAP1 downstream targets in mice liver tissues.
(A) The expression of NF2, YAP1 and p-YAP1 were detected by WB. GAPDH was used as a normalized control. (B) Relative mRNA expression of *Ctgf*, *Ccnd1* and *Cyr61* from *Trim65* cKO and wildtype mice liver tissues after *YAP1*-WT or *YAP1*-5SA retrieved measured by RT-qPCR. Data are presented as mean ± SD. *** ($p < 0.001$), ** ($p < 0.01$), * ($p < 0.05$). One-way ANOVA for multiple comparisons.

Supplemental Table S3. List of siRNAs utilized in this study.

Gene Name	Sequence
siNC	UUCUCCGAACGTGTCACGUUU
si*TRIM65*-1	CCGGUCCCAAGCACAGUUU
si*TRIM65*-2	GGCUCCUGGGCAUGGAUUU
si*OGT*-2	GCUGAGCAGUAUUCCGAGAAA
si*OGT*-3	GCCCUAAGUUUGAGUCCAAAU
si*YAP1*	CAGGUGAUACUAUCAACCAAA

Supplementary Table S4. List of primers utilized in RT-qPCR

Gene Name	Forward	Reverse
18S rRNA	CAGCCACCCGAGATTGAGCA	TAGTAGCGACGGGCGGTGTG
Human-TRIM65	AAGCAGCCAGATCCAGAACTC	CTCAGTGCTGTCGTGTGCT
Human-CTGF	CAGCATGGACGTTCGTCTG	AACCACGGTTTGGTCCTTGG
Human-UMPS	GGACTACACTAGAGCAGCGG	TGGCCAAGATTATCTCCTCCTG
Human-CYR61	GGTCAAAGTTACCGGGCAGT	GGAGGCATCGAATCCCAGC
Human-CCND1	CAATGACCCCGCACGATTTC	CATGGAGGGCGGATTGGAA
Human-YAP1	TAGCCCTGCGTAGCCAGTTA	TCATGCTTAGTCCACTGTCTGT
Human-UCK1	AGTTGCTGGGACAGAACGAG	CTGCCGTCAGGACCTTGTAG
Human-UCK2	CTGAGCCAGGATAGCTTCTACC	CATACACGGGGATCTGGACTG
Human-NME6	TCCAGCTCACTCTAGCCCTG	CGGTAAAACCTCTGGCAATCTT
Human-UPP1	TGATTGCCCCGTCAGACTTTT	CACCAACGCACCTGATGAAG
Human-UPP2	GCCTCCAATAGGTCCATGAGA	ACATTGCTGGTAGGTTGTGTG
Human-UAP1	AATGACCTCAAACTCACGTTGT	GCTCTGCATAAAGTTCTACCTGT
Human-UAP1L1	CCAACGTGGTCATGTTTGAGC	GGATGTTGTCCACACAGTACAC
Human-CMPK1	GGAAGGCAGATGTATCTTTCGTT	TGTTGACTGAAGGTAGGTCTGA
Human-CMPK2	CCAGGTTGTTGCCATCGAAG	CAAGAGGGTGGTGACTTTAAGAG
Human-CD206	GTGATGGGACCCCTGTAACG	CTGCCCAGTACCCATCCTTG
Human-CD163	TTTGTCAACTTGAGTCCCTTCAC	TCCCGCTACACTTGTTTTCAC
Human-TGFB1	GGCCAGATCCTGTCCAAGC	GTGGGTTTCCACCATTAGCAC
Human-IL10	GCTCCTGCCCTTAGGGTTAC	GAAGAAATCGATGACAGCGCC
Mouse-Trim65	AGGAAGAAGCTCTGGCAGAATTA	CCAGCTTTTGGCGTGATAGC
Mouse-Ctgf	GGGCCTCTTCTGCGATTTC	ATCCAGGCAAGTGCATTGGTA
Mouse-Umps	GTCACCGAGCTGTATGACGTG	GGTAACGCTGTATAAGGAACTCC
Mouse-Cyr61	CTGCGCTAAACAACTCAACGA	GCAGATCCCTTTCAGAGCGG
Mouse-Ccnd1	GCGTACCCTGACACCAATCTC	CTCCTCTTCGCACTTCTGCTC
Mouse-Actb	GGCTGTATTCCCCTCCATCG	CCAGTTGGTAACAATGCCATGT
CHIP-*UMPS-R1*	ACGGGGTTTCACCATGTTAGCCA	CTCACGCCTGTAATCCCAGCA
CHIP-*UMPS-R2*	GAGAAGCACAAACTGGCGCT	GATGACGTCACCCGCGAAGT
CHIP-*FASN*	AAACCACCGCCCCCCGACTT	GGGTAGTCCCCAGTGTGGCCCA

Supplementary Table S5. Antibodies utilized for (IP-)WB and IF assays.

Antibody	Catalogue Number	Company	Species	WB Dilution	IP Dilution	IF Dilution
OGT	66823-1-Ig	Proteintech	Mouse	1:2000	/	1:500
TRIM65	HPA021578	Atlas Antibodies	Rabbit	1:1000	/	1:500
GAPDH	5174	Cell Signaling Technology	Rabbit	1:1000	/	/
GAPDH	60004-1-Ig	Proteintech	Mouse	1:50000	/	1:500
Flag-Tag	M20008	Abmart	Mouse	1:5000	1:200	/
HA-Tag	3724	Cell Signaling Technology	Rabbit	1:1000	1:100	/
O-GlcNAc	ab2739	Abcam	Mouse	1:1000	/	/
O-GlcNAc	PTM-951RM	PTMbio	Rabbit	1:1000	1:100	/
NF2	21686-1-AP	Proteintech	Rabbit	1:1000	1:100	1:250
Myc-Tag	M20002	Abmart	Mouse	1:5000	1:200	/
K63-Ub	05-1308	Sigma-Aldrich	Rabbit	1:1000	/	/
LATS1	3477	Cell Signaling Technology	Rabbit	1:1000	/	/
p-LATS1	8654	Cell Signaling Technology	Rabbit	1:1000	/	/
YAP1	ab52771	Abcam	Rabbit	1:1000	/	1:500
YAP1	13584-1-AP	Proteintech	Rabbit	1:1000	5μl (CHIP)	/
CREB	9197	Cell Signaling Technology	Rabbit	1:1000	5μl (CHIP)	/
p-YAP1	4911	Cell Signaling Technology	Rabbit	1:1000	/	/
Histone H3	17168-1-AP	Proteintech	Rabbit	1:2000	/	/
c-Met	25869-1-AP	Proteintech	Rabbit	1:1000	/	/
β-catenin	M24002	Abmart	Mouse	1:1000	/	1:500

Supplementary Table S6. Antibodies utilized for flow cytometry.

Antibody	Clone	Catalogue Number	Company	Fluorescence	Species	Dilution
Anti-Mouse CD3e	145-2C11	563565	BD Biosciences	BUV395	Hamster	1:200
Anti-Mouse CD8a	53-6.7	566409	BD Biosciences	BB700	Rabbit	1:200
Anti-Mouse CD4	GK1.5	563331	BD Biosciences	BUV786	Rabbit	1:200
Anti-CD11b	M1/70	563015	BD Biosciences	BV605	Rabbit	1:200
Anti-Mouse F4/80	T45-2342	565411	BD Biosciences	BV421	Rabbit	1:200
Anti-Mouse CD206	Y17-505	568273	BD Biosciences	PE	Rabbit	1:200
Anti-Mouse NK1.1	PK13b	550627	BD Biosciences	APC	Mouse	1:200
Anti-Mouse CD45	30-F11	11-0451-81	eBioscience	FITC	Rabbit	1:200
Anti-Mouse CD16/32	2.4G2	70-0161-U500	TONBO	/	/	1:100
